# Therapeutic potential of 1,3,4-oxadiazoles as potential lead compounds for the treatment of Alzheimer's disease†

**DOI:** 10.1039/d3ra01953e

**Published:** 2023-06-09

**Authors:** Saira Naseem, Ahmed Temirak, Aqeel Imran, Saquib Jalil, Shamool Fatima, Parham Taslimi, Jamshed Iqbal, Mussarat Tasleem, Muhammad Nawaz Tahir, Zahid Shafiq

**Affiliations:** a Institute of Chemical Sciences, Bahauddin Zakariya University Multan 60800 Pakistan zahidshafiq@bzu.edu.pk; b National Research Centre, Chemistry of Natural and Microbial Products Department, Pharmaceutical and Drug Industries Research Institute Dokki, Cairo P.O. Box 12622 Egypt; c Centre for Advanced Drug Research, COMSATS University Islamabad, Abbottabad Campus Abbottabad-22060 Pakistan drjamshed@cuiatd.edu.pk; d Department of Pharmacy, COMSATS University Islamabad, Lahore Campus Punjab 54000 Pakistan; e Department of Biotechnology, Faculty of Science, Bartin University 74100 Bartin Turkey; f Department of Physics, University of Sargodha Sargodha Pakistan; g Department of Chemistry, COMSATS University Islamabad, Abbottabad Campus Abbottabad-22060 Pakistan

## Abstract

Monoamine oxidase and cholinesterase enzymes are important targets for the treatment of several neurological diseases especially depression, Parkinson disease and Alzheimer's. Here, we report the synthesis and testing of new 1,3,4-oxadiazole derivatives as novel inhibitors of monoamine oxidase enzymes (MAO-A and MAO-B) and cholinesterase enzymes (acetyl and butyryl cholinesterase (AChE, BChE). Compounds 4c, 4d, 4e, 4g, 4j, 4k, 4m, 4n displayed promising inhibitory effects on MAO-A (IC_50_: 0.11–3.46 μM), MAO-B (IC_50_: 0.80–3.08 μM) and AChE (IC_50_: 0.83–2.67 μM). Interestingly, compounds 4d, 4e and 4g are multitargeting MAO-A/B and AChE inhibitors. Also, Compound 4m displayed promising MAO-A inhibition with IC_50_ of 0.11 μM and high selectivity (∼25-fold) over MAO-B and AChE enzymes. These newly synthesized analogues represent promising hits for the development of promising lead compounds for neurological disease treatment.

## Abstract 2

Monoamine oxidase and cholinesterase enzymes are important targets for the treatment of several neurological diseases especially depression, Parkinson disease and Alzheimer's.

## Graphical abstract

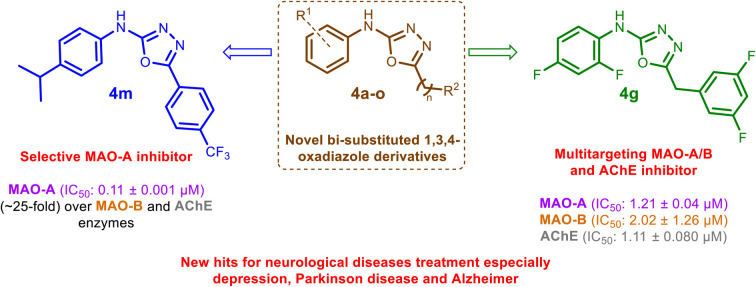

## 1. Introduction

Mental and neurological disorders are often highly disabling and are associated with increased premature mortality rates. Other than the health consequences, these disorders have also their negative impacts on society and the economy. There are many reported neurological disorders including; Parkinson's disease, Alzheimer's disease, stroke, epilepsy, migraine, meningitis, spinal cord injuries and many others.^1^

Alzheimer's disease (AD) is a complicated neurological disorder.^2^ The number of AD patients worldwide in 2019 was around 57 million and it is expected that this number will triple by 2050. Many hypotheses have been reported explaining the onset and progression of AD pathology including cholinergic neuron damage, inflammation, oxidative stress and the abnormal deposition of amyloid β (Aβ) protein in the neurons, *etc.*^3^ The two major hallmarks of AD diagnosis are β-amyloid peptide (Aβ) and the generation of neurofibrillary tangles of the axon-enriched microtubule-associated protein tau.^4^

The brain can only function well when there is an equilibrium of the neurotransmitter systems for example: acetylcholine (ACh), dopamine, gamma-aminobutyric acid (GABA), serotonin, and others.^5^ Monoamine oxidase enzyme (MAO) is a flavin-containing membrane-bounded enzyme located particularly in brain and liver.^6^ It catalyzes the endogenous and exogenous oxidative deamination of monoamine neurotransmitters resulting in the formation of hydrogen peroxide which is a negotiator of oxidative stress.^5^ This affects the concentration of many xenobiotic and neurotransmitter amines leading to several neurological diseases.^7^

Two distinct forms of monoamine oxidase (MAO-A and MAO-B) are present in most tissues of mammals and displays different structure, regulation, and function. MAO-A mainly deaminates neurotransmitters such as serotonin, adrenaline and noradrenaline which are aromatic in nature. MAO-B which is the main isoform located in the brain preferentially oxidizes benzylamines, β-phenylethylamine (PEA) and polyamines.^8^ Tryptamine and dopamine which are basically dietary amines are generally effected by both enzyme isoforms.^9^ MAO plays an important role in the progression of several neurological diseases including AD. They increase the amyloid-beta (Aβ) deposition, impair the cognitive functions as a result of neuronal loss and causing the generation of neurofibrillary tangles. Therefore, monoamine oxidase inhibitors (MAOIs) are widely used in the treatment of several neurological and psychiatric conditions.^10^

Cholinesterase enzymes (ChEs) are involved by the degradation of the neurotransmitter acetyl choline (ACh) in the brain. The decrease in Ach levels in brain is involved with the cognitive dysfunction and memory loss in AD patients. Two ChEs are reported, acetylcholinesterase (AChE) and butyrylcholinesterase (BChE).^11^ AChE are more expressed than BChE in the cerebral cortex and in the hippocampus of the brain, whereas, during the progress of AD, a great increase in the activity of BChE was reported.^12^ Based on the important roles of cholinesterase enzymes in the pathophysiology of AD, their inhibitors (AChE and BuChE inhibitors) are FDA approved for the symptomatic treatment of AD.^13^

**Fig. 1:**
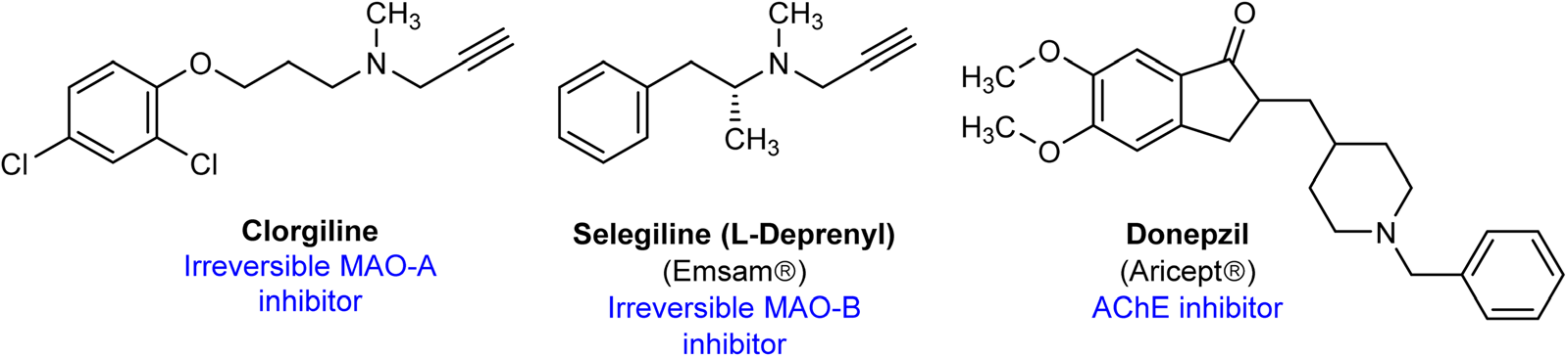
Chemical structure of several reported MAO and AChE inhibitors.

Several MAO inhibitors were approved by FDA for the treatments of several neurological disorders or psychiatric diseases.^6^ The irreversible MAO-A/B inhibitor tranylcypromine is used as antidepressant. Similarly, the highly selective and irreversible MAO-A inhibitor clorgyline demonstrated antidepressant effects in human, however, it is not used clinically because of dietary interactions (Fig. 1).^14^l-Deprenyl, the irreversible MAO-B inhibitor is approved for the treatment of Parkinson disease with trade name Selegiline.^15^ Currently, several cholinesterase inhibitors, for example donepezil, neostigmine, galantamine and others, are used for the treatment of AD, myasthenia gravis and other disorders.^13^

**Fig. 2:**
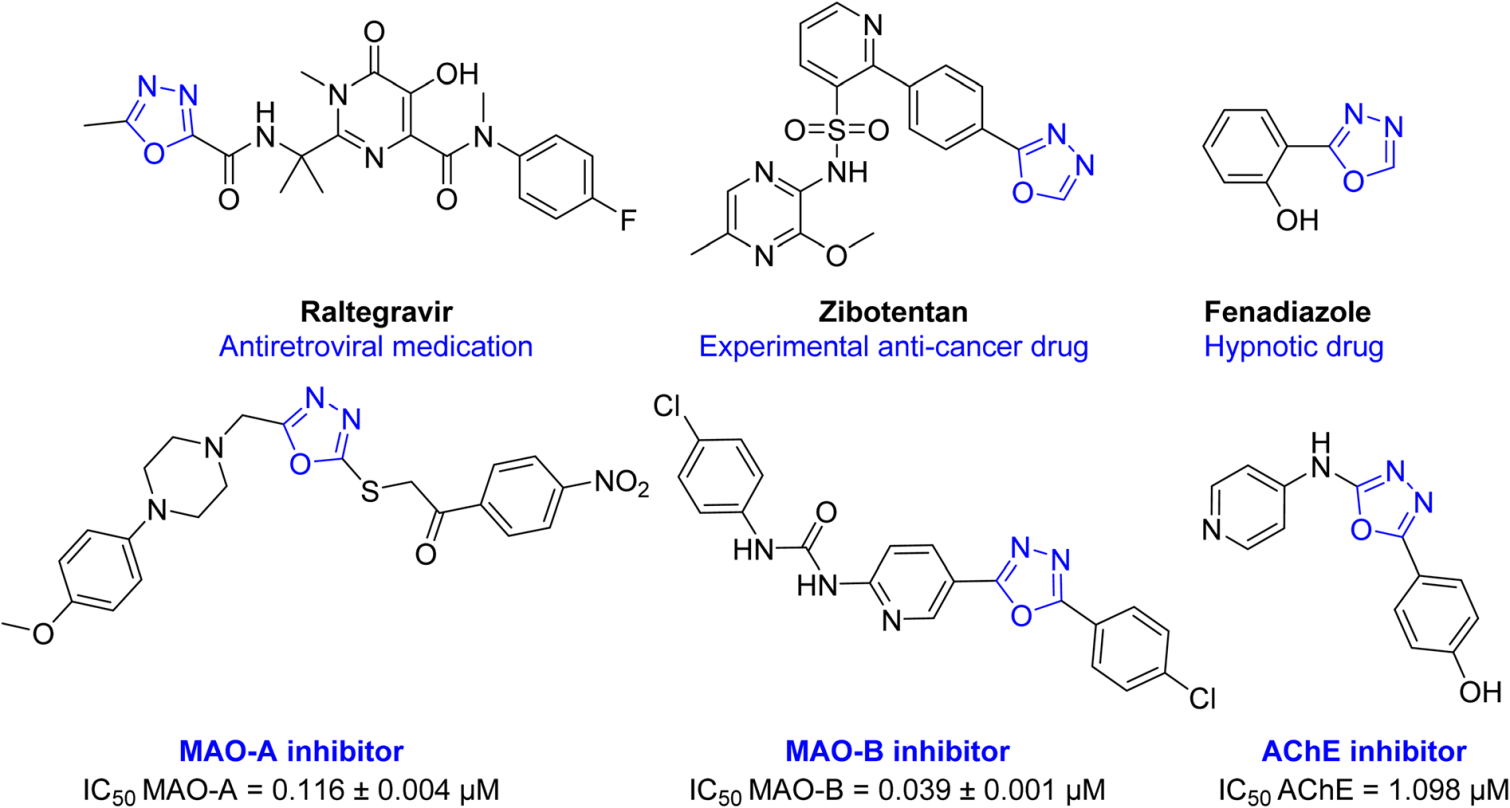
Chemical structure of some drugs and test compounds having 1,3,4-oxadiazole ring in their structure.

Oxadiazole ring is a highly versatile building block in several medicines and in the scope of future drug development.^16^ Several activities were reported with 1,3,4-oxadiazole containing compounds including; anti-hypertensive (Tiodazosin), anti-inflammatory, anti-fungal, antibacterial, antiviral (Raltegravir), anticonvulsant, hypnotic (Fenadiazole), anticancer (Zibotentan), anti-AD, and many others.^7,16–21^ Also, several oxadiazole derivatives were reported in literature as MAO and ChE inhibitors (Fig. 2).^9,22–25^

In the light of the above discussion, oxadiazole is a versatile ring involved in several MAO and ChE inhibitors. We herein report the synthesis of a new library of 1,3,4-oxadiazol analogues using unique and effective synthetic strategies that resulted in structural diversity of unsymmetrical aryl/alkyl-substitutions. These new compounds were further evaluated against monoamine oxidase (MAO-A and MAO-B) and cholinesterase (AChE and BChE) enzymes. To validate the results, *in silico* docking studies have been conducted to assess the binding interaction of the synthesized compounds inside the active site of MAO- and ChE enzymes.

## 2. Results and discussion

### 2.1 Chemistry

**Scheme 1:**
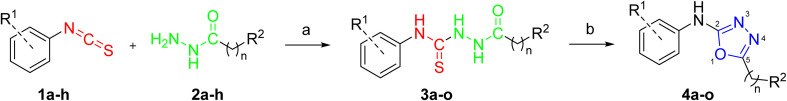
Synthesis of 1,3,4-oxadiazole derivatives 4a–o. Reagents and conditions: (a) benzene, 80 °C, 6 h; (b) HgCl_2_, TEA, DMF, rt, 8 h, 71–83%.

The synthesis of the target compounds 4a–o is outlined in Scheme 1. We started with the synthesis of carbothioamides 3a–o through the reaction of various isothiocyanates 1a–h with acid hydrazides 2a–h.^26^ The cyclization of carbothioamides 3a–o using mercuric chloride as a catalyst and triethylamine as a base afforded the 1,3,4-oxadiazole derivatives 4a–o (Table 1).^18^ The procedure as previously reported involves desulfurization of thiosemicarbazides followed by cyclization reactions using mercuric chloride to obtain the desired oxadiazole derivatives.^20^

**Table 1:** A list of the synthesized oxadiazole derivatives 4a–o

Compound	R^1^	R^2^	*n*	Yield (%)
4a	3-Cyano	4-Trifluorophenyl	0	83
4b	3-Cyano	2-Trifluromethylbenzyl	1	78
4c	2-Fluoro	2-Trifluromethylbenzyl	1	72
4d	H	4-Pyridyl	0	80
4e	2,4-Dimethyl	3-Fluorophenyl	0	73
4f	2-Fluoro	3,5-Difluorobenzyl	1	71
4g	2,4-Difluoro	3,5-Difluorobenzyl	1	82
4h	2-Fluoro	2-Fluoro-5-methylphenyl	0	75
4i	2-Fluoro	4-Trifluoromethylphenyl	0	74
4j	3-Cyano	4-Pyridyl	0	75
4k	2,4-Dimethyl	4-Pyridyl	0	75
4l	4-Bromo	4-Trifluoromethylphenyl	0	74
4m	4-Isopropyl	4-Trifluoromethylphenyl	0	73
4n	2-Methyl	Phenyl	0	83
4o	H	3-Fluoro-2-methyl	0	81

The progress of the reaction was monitored by thin layer chromatography (TLC). Upon completion of the reaction, chloroform was added to the reaction mixture and the suspension was filtered and washed carefully to remove the side product mercuric sulfide. The obtained compounds were recrystallized from dichloromethane and their structure and purity were confirmed by different spectroscopic methods including; nuclear magnetic resonance (NMR), infrared (IR), mass spectroscopy and elemental analysis.

### 2.2 Single crystal X-ray analysis

**Fig. 3:**
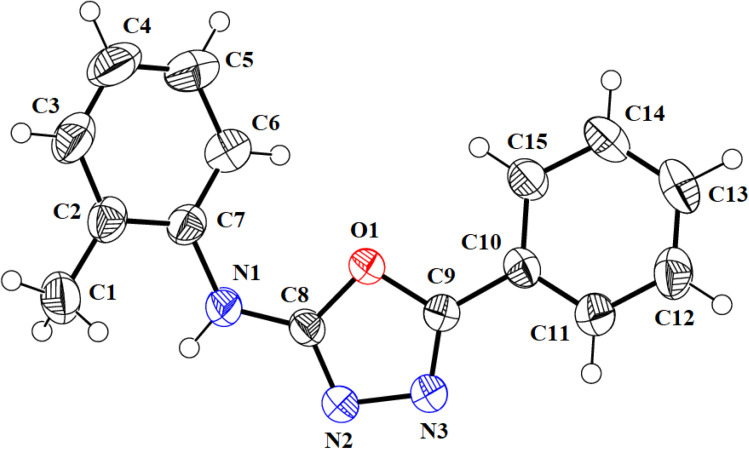
ORTEP diagram of compound 4n that is drawn at probability level of 50%. H-atoms are shown by small circles of arbitrary radii.

**Fig. 4:**
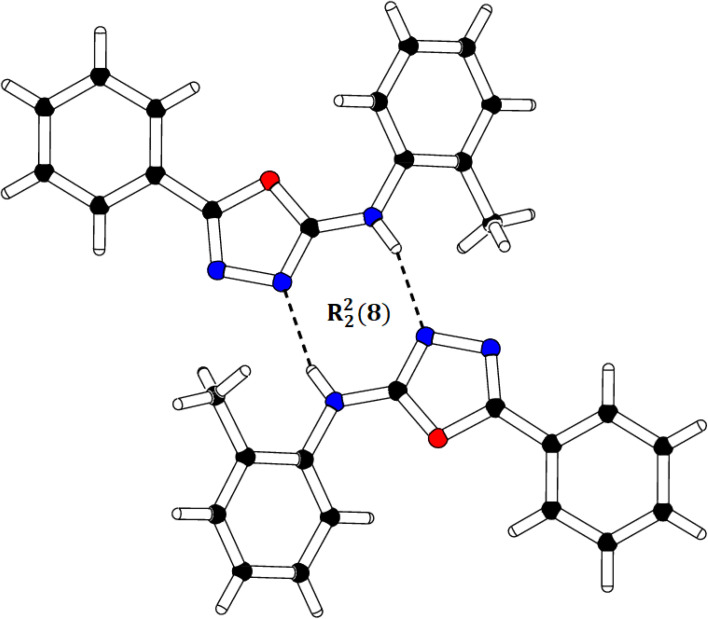
Packing diagram of compound 4n showing the dimerization between two 4n molecules.

We successfully resolved the X-ray structure of compound 4n (*N*-(2-methylphenyl)-5-phenyl-1,3,4-oxadiazol-2-amine) using Bruker Kappa Apex-II CCD diffractometer having Mo-Kα X-rays source as depicted in Fig. 3. The *o*-toluidine ring A (C1–C7/N1), 1,3,4-oxadiazole ring B (C8/C9/N2/N3/O1) and attached phenyl ring C (C10–C15) are planar with root mean square deviation of 0.0060, 0.0037 and 0.0101 Å, respectively. The dihedral angles A/B, A/C and B/C are 38.1 (4)°, 39.7 (4)° and 15.3 (7)°, respectively (please check ESI file for further details displayed in Table S1†). Hence, the overall molecule is non-planar. The molecules are connected with the form of dimers through N–H⋯N bonding to form R^2^_2_ (8) loop as shown in Fig. 4 and given in Table S2.† The acceptor N-atom is that of nearest to NH group *i.e.*, N2. The crystal packing is further stabilized by C–H⋯π and π⋯π stacking interactions.

### 2.3 Biological activities

#### 2.3.1 Inhibition of MAO-A and B, acetyl cholinesterase (AChE) and butryl cholinestrase (BChE)

Newly synthesized oxadiazole derivatives were tested for their potential inhibition against monoamine oxidase A and B, acetyl cholinesterase (AChE) and butyryl cholinesterase (BChE) (Table 2). Our compounds are sharing the versatile 1,3,4-oxadiazol-2-amine core which is present in several previously reported MAO and ChE inhibitors.^9,22,25^ The 1,3,4-oxadiazol-2-amine is involved with several key interactions within the active site of MAO and ChE enzymes as elucidated in the molecular docking section. Substitutions on the 2-amino and on the C5 positions of the oxadiazole ring with different aryls along with different linkers length contributed to the different biological activities of our compounds.

**Table 2:** Inhibitory effects of compounds 4a–o against MAO-A, MAO-B, AChE and BChE

Compound	MAO-A	MAO-B	AChE	BChE
	aIC_50_ ± SEM^b^ (μM)/% age inhibition			
4a	33.23^c^	37.06^c^	42.32%^c^	20.54%^c^
4b	2.86 ± 1.51^b^	35.73^c^	31.34%^c^	23.65%^c^
4c	1.90 ± 0.25^b^	0.80 ± 0.27^c^	36.12%^c^	14.76%^c^
4d	1.44 ± 0.22^b^	1.04 ± 0.22^c^	0.83 ± 0.007^a^	23.67%^c^
4e	1.47 ± 0.45^b^	1.91 ± 0.89	1.96 ± 0.176^a^	10.54%^c^
4f	34.82^c^	39.46^c^	2.03 ± 0.434^a^	22.61%^c^
4g	1.21 ± 0.04^b^	2.02 ± 1.26	1.11 ± 0.080^a^	15.23%^c^
4h	21.87^c^	29.36^c^	1.51 ± 0.462^a^	12.43%^c^
4i	38.11^c^	32.53^c^	37.12%^c^	21.45%^c^
4j	1.02 ± 0.09^b^	18.57^c^	1.56 ± 0.309^a^	20.55%^c^
4k	3.46 ± 2.58^b^	1.10 ± 1.16	39.65%^c^	14.67%^c^
4l	36.81^c^	24.86^c^	44.56%^c^	26.91%^c^
4m	0.11 ± 0.001^b^	3.08 ± 0.06^c^	2.67 ± 0.029^a^	15.78%^c^
4n	21.07^c^	18.47^c^	1.23 ± 0.969^a^	28.87%^c^
4o	29.98^c^	22.65^c^	2.32 ± 0.080^a^	32.98%^c^
Clorgyline^d^	0.005 ± 0.03	61.35 ± 1.13	—	—
Deprenyl^d^	67.25 ± 1.02	0.019 ± 0.01	—	—
Donepezil^d^	—	—	0.032 ± 0.003^a^	6.41 ± 0.34^a^

a IC_50_ half-maximal half inhibitory concentration in micromolar range.

b SEM standard error mean.

c %age inhibition.

d Positive control.

It was observed that several compounds exhibited strong inhibition against MAO-A/MAO-B and AChE, however, all the synthesized compounds did not show strong inhibition against BChE. Among the tested compounds 4c, 4d, 4e, 4g, 4j, 4k and 4m displayed potent inhibition against MAO-A with IC_50_ in lower micromolar range. Compound 4m exhibited potent inhibition against MAO-A with IC_50_ value of 0.11 μM whereas 4a, 4f, 4h, 4i, 4l and 4n showed weak inhibition against MAO-A. Inhibitory potential of the synthesized derivatives against MAO-B elaborated in Table 2 which displayed that compounds 4c, 4d, 4e, 4g, 4k and 4m exhibited strong inhibition against MAO-B with IC_50_ value in sub micromolar range, amongst these 4c exhibited potent inhibition of MAO-B with IC_50_ value of 0.80 μM.

The majority of tested compounds against AChE have shown potent inhibition with IC_50_ values in lower micromolar range. The compounds 4d, 4e, 4f, 4h, 4i, 4j, 4m and 4n and exhibited potent inhibition against AChE, amongst all identified inhibitors 4d was promising inhibitor with IC_50_ value of 0.83 μM. On the contrary, compounds 4a, 4b, 4c, 4i, 4k and 4l exhibited moderate to weak inhibition of AChE. However, all the tested compounds have shown weak inhibition against BChE (Table 2).

Substitution on the phenyl ring of the aniline moiety with bulky groups such as bromo as in compound 4l demolished the activity towards all MAO and ChE enzymes. On the other hand, replacement of the bromo with the isopropyl group as in compound 4m increased dramatically the activity and the selectivity towards the MAO-A enzyme with IC_50_ value of 0.11 μM. Compound 4d having the terminal unsubstituted phenyl and pyridyl rings displayed promising inhibitory effects against MAO-A, MAO-B and AchE enzymes. Upon substitution on the phenyl ring with *m*-cyano group as in compound 4j, the compound lost its inhibitory effects on MAO-B. On another example, having 2.4-dimethyl groups on the phenyl ring as in compound 4k, the compound restored its inhibitory effects on MAO-B, however, it lost its inhibitory effects on AchE. From these examples, we can conclude that small structural modifications can have great impact on the inhibitory effects of the developed compounds. Also, having multi-targeting compounds such as compounds 4d, 4e and 4g would be an interesting starting point for development of new chemical entities.

### 2.4 Molecular docking studies

#### 2.4.1 Docking studies on MAO-A and MAO-B enzymes

**Fig. 5:**
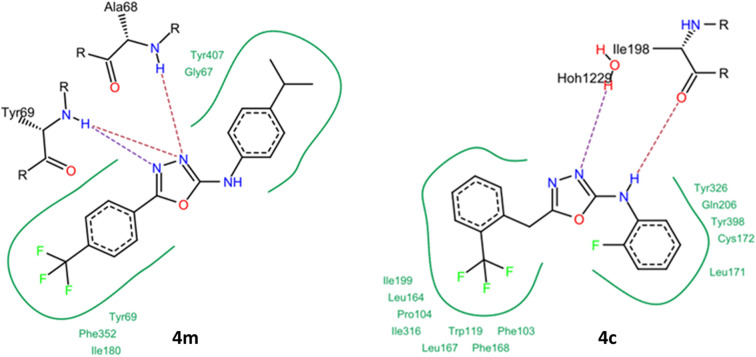
(1) Compound 4m docked at the active site of MAO-A enzyme and its 2D diagram. (2) Compound 4c docked at active site of MAO-B with its 2D diagram representing interactions of amino acids residues with active site.

Newly synthesized compounds were tested against monoamine oxidase A for the *in vitro* inhibition and molecular docking studied were performed for the identified inhibitor of Monoamine oxidase A/B. The experimental results were correlated with *in silico* studies and interactions of amino acid residues of the active pocket with ligand were studied. The compound 4m exhibited strong hydrogen bond interaction with amino acid residues Tyr69, Ala68 of active site of enzyme. The hydrogen atom of the Ala68, Tyr69 made hydrogen bond with two nitrogen atom of oxadiazole moiety of compound 4m (Fig. 5) whereas hydrophobic interactions involved the various amino acids residues with phenyl ring. The hydrophobic interactions include π–π/π-alkyl interactions, were displayed by phenyl ring of the ligand *vs.* amino acid residues Tyr407, Gly67, Tyr69, Ile80 and Phe32 (Fig. 5 2D interactions). The compounds 4c exhibited IC_50_ value in sub micromolar range against MAO-B therefore it was selected to assess its binding affinity with the active pocket of monoamine oxidase B. The strong hydrogen bonding of 4c with active site originated due to interactions of Ile198 and water molecule 1229. The trifluoromethyl moiety and phenyl ring of 4c exhibited hydrophobic interactions with amino acids residues: Ile99, Leu64, Pro104, Ile316, Leu67, Trp219, Phe168 and Phe103 of active pocket whereas fluoro-substituted benzene ring displayed van der Waal interactions with active site residues *i.e.*, Tyr326, Gln206, Tyr298, Cys172 and Leu 171 (Fig. 5).

#### 2.4.2 Docking studies on AChE enzyme

**Fig. 6:**
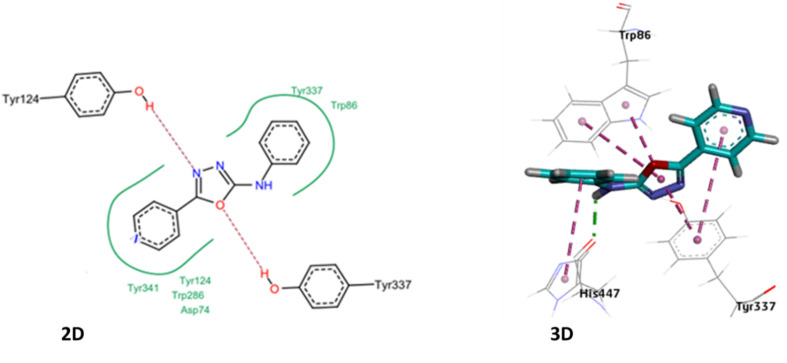
(1) Two-dimensional docked pose of compound 4d at the active pocket of AChE. (2) Three-dimensional docked pose of compound 4d with active pocket of AChE.

The newly synthesized compounds were also tested against acetyl cholinesterase. Among the tested compounds, 4d was most promising inhibitor of AChE with IC_50_ value of 0.83 μM. The binding affinity of 4d was analyzed through performing molecular docking studies where the best docked pose was further explored for its interaction with active pocket. The anchoring of 4d into the cleft of active pocket was mainly associated with strong hydrogen bonding and some van der Waals interactions with the residues of the active site of AChE. The nitrogen atom of oxadiazole ring of 4d exhibited hydrogen bond between –OH group of Tyr124 residue of active site whereas the oxygen atom of oxadiazole ring have displayed hydrogen bonding with –OH group of Tyr337. The hydrophobic interactions are displayed by pyridine and benzene ring with the residues like Tyr341, Tyr214, Trp286, Asp74, Try337 and Trp86 of the active pocket of AChE (Fig. 6).

### 2.5 *In silico* pharmacokinetic analysis and computational toxicological evaluation

**Fig. 7:**
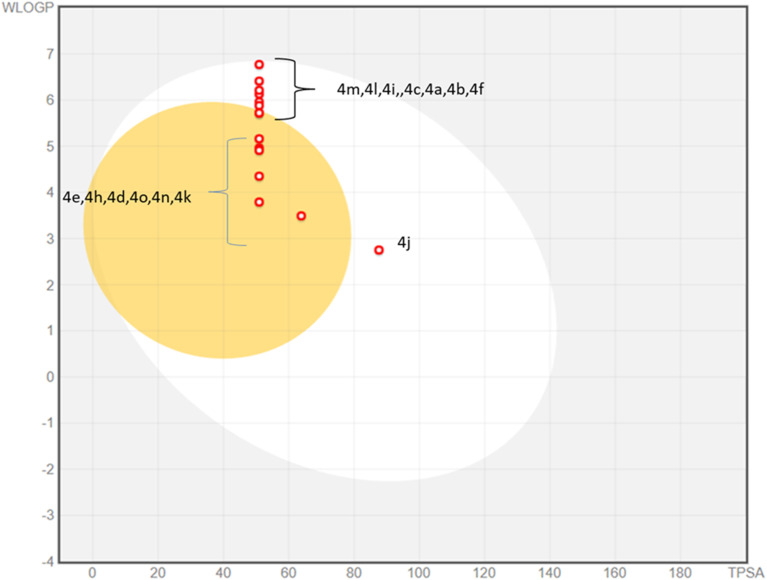
Boiled-egg plot for synthesized derivatives 4a–o.

The physicochemical properties and drug likeness were determined through SwissADME web server. Newly synthesized compounds exhibited strict compliance towards rule of Lipinski. The boiled egg plot displayed prominent two portions; white part depicted the good gastrointestinal absorption properties of the tested compounds whereas yellow yolk of the boiled egg showed the permeation ability to blood–brain barrier. Among 4a–o compounds, majority of compounds have displayed excellent gastrointestinal absorption properties whereas some of them (4e, 4h, 4d, 4o, 4n, 4k) exhibited blood–brain barrier permeation ability (Fig. 7). None of the 4a–o compounds exhibited Lipinski violation. Due to their druggable nature these compounds were tested against monoamine oxidase A and B in search of new leads to treat Alzheimer diseases. The detailed parameters for *in silico* pharmacokinetic are shown in the Table 3.

**Table 3:** *In silico* determination of pharmacokinetic profiles using SwissADME server

No.	MW	H-bond acceptor	H-bond donors	TPSA	WLOGP	GI absorption	BBB permeant	Lipinski #violations	PAINS #alerts
4a	319.28	6	1	50.95	5.96	High	No	0	0
4b	333.31	6	1	50.95	5.88	High	No	0	0
4c	337.27	7	1	50.95	6.13	High	No	1	0
4d	297.33	4	1	50.95	4.96	High	Yes	0	0
4e	291.23	6	1	50.95	5.16	High	Yes	0	0
4f	309.22	7	1	50.95	5.72	High	Yes	1	0
4g	309.22	7	1	50.95	5.72	High	Yes	1	0
4h	287.26	5	1	50.95	4.91	High	Yes	0	0
4i	323.25	7	1	50.95	6.21	High	No	0	0
4j	263.25	5	1	87.63	2.75	High	No	0	0
4k	266.3	4	1	63.84	3.49	High	Yes	0	0
4l	384.15	6	1	50.95	6.41	High	No	1	0
4m	347.33	6	1	50.95	6.77	High	No	1	0
4n	251.28	3	1	50.95	3.79	High	Yes	0	0
4o	269.27	4	1	50.95	4.35	High	Yes	0	0

In the early phases of drug discovery and lead optimization, computational toxicity parameters cab be predicted through various software. In present study, a software named ProTox-II – Prediction of Toxicity of Chemicals was applied to determine toxicity of the compounds (https://tox-new.charite.de/protox_II/index.php?site=home). The prediction of various toxicity end points like cytotoxicity, immunogenicity, carcinogenicity, mutagenesis were computed for the compounds 4a–o (Table S3†). Among the tested compounds, majority of the compounds exhibited toxicity class 4 and could be considered as druggable compounds. The predicted median lethal dose (LD_50_) for the 4i and 4o was 1190 mg kg^−1^ whereas 4b, 4c have shown 800 mg kg^−1^. All other compounds exhibited LD_50_ above 500 mg kg^−1^ which corresponds with toxicity class 4. Among the toxicity classes, class I, II(fetal), whereas class III considered as toxic. However, class IV and class V may be considered as harmful but class VI belongs to non-toxic chemicals (https://www.osha.gov/hazcom). All the tested compounds exhibited no activity for the mutagenicity, carcinogenicity, immunogenicity, hence these compounds could further be explored for the treatment of Alzheimer's disease. Details for the predicted toxicity parameters can be seen in the ESI.†

## 3. Conclusion

A series of *N*-phenyl-5-aryl-1,3,4-oxadiazol-2-amine derivatives were synthesized bearing different substituents. Compounds 4a–o were further tested against monoamine oxidase enzymes (MAO-A and MAO-B), cholinesterase enzymes (acetylcholine esterase (AChE) and butyryl cholinesterase (BChE)). All compounds showed promising inhibition in the lower micro molar range against the four target enzymes. Several compounds showed promising inhibitory effects on MAO-A (IC_50_: 0.11–3.46 μM), MAO-B (IC_50_: 0.80–3.08 μM) and AChE (IC_50_: 0.83–2.67 μM). Remarkably, compounds 4d, 4e and 4g are multitargeting MAO-A/B and AChE inhibitors. Compound 4m displayed promising inhibition against MAO-A enzyme with IC_50_ value of 0.11 μM and high selectivity (∼25-fold) over MAO-B and AChE enzymes. Furthermore, the docking studies further open the window for binding site interactions of the inhibitors with the four studied enzymes. These newly developed multi-targeting compounds represent promising hits for the future treatments of neurological diseases.

## 4. Experimental

### 4.1 Chemistry

The crystal data was collected on Bruker Kappa Apex-II CCD diffractometer having Mo-Kα X-rays source. SHEXT-2014 and SHELXL-2019/2 softwares were used for the structure solution and refinement, respectively. Anisotropic displacement parameters were assigned to the non-hydrogen atoms whereas isotropic displacement parameters were assigned to hydrogen atoms. The H-atoms are placed by using riding model. ORTEP-III, PLATON software were used for the graphical representations.

### 4.2 General procedure for the synthesis of 1,3,4-oxadiazol derivatives (4a–o)

In a round-bottom flask, a suspension of carbothioamides (3a–o, 1.0 mmol) and mercuric chloride (1.25 mmol) were dispersed in dry DMF (5 mL), and a catalytic amount of triethylamine (0.1 mmol) was added dropwise. The reaction mixture was stirred at rt for 8 hours. Upon completion of the reaction, the mixture was filtered under reduced pressure and washed with 20 mL chloroform. The filtrate was extracted with distilled water (3× 10 mL), then the organic layers were collected, dried over MgSO_4_ and concentrated under vacuum. The obtained residues were further dissolved in dichloromethane and recrystallized to obtain nice powder of the products 4a–o.

#### 3-((5-(4-(Trifluoromethyl)phenyl)-1,3,4-oxadiazol-2-yl)amino)benzonitrile (4a)

Color: off-white; yield: 83%, M.P: 220–223 °C; IR *υ*_max_ (cm^−1^): 3362 (N–H), 1635 (C=N); ^1^H-NMR (500 MHz, DMSO-*d*_*6*_) *δ* 2.09 (s, 3H, C*H*_3_), 7.51 (d, 1H, *J* = 8.0 Hz, Ar–*H*), 7.61 (t, 1H, *J* = 8.0 Hz, Ar–*H*), 7.88 (dd, 1H, *J* = 1.2, 7.6 Hz, Ar–*H*), 7.95 (d, 2H, *J* = 8.4 Hz, Ar–*H*) 8.04 (m, 1H, Ar–*H*), 8.10 (d, 2H, *J* = 8.0 Hz, Ar–*H*), 11.27 (s, 1H, Ar-N*H*); ^13^C-NMR (125 MHz, DMSO-*d*_*6*_) *δ* 31.15, 112.42, 119.18, 120.10, 126.08, 126.84, 126.91, 127.84, 131.15, 139.80, 157.65, 160.36, 207.02. Anal. calcd. for C_16_H_9_F_3_N_4_O (330.26): C, 60.19; H, 3.79; N, 13.66; found: C, 60.29; H, 3.55; N, 13.49.

#### 3-((5-(2-(Trifluoromethyl)benzyl)-1,3,4-oxadiazol-2-yl)amino)benzonitrile (4b)

Color: off-white; yield: 78%, M.P: 153–157 °C; IR *υ*_max_ (cm^−1^): 3366 (N–H), 1650 (C=N); ^1^H-NMR (500 MHz, DMSO-*d*_*6*_) *δ* 2.09 (s, 3H, C*H*_3_), 4.38 (s, 2H, C*H*_2_), 7.43–7.46 (m, 1H, Ar–*H*), 7.53–7.60 (m, 3H, Ar–*H*), 7.69–7.71 (m, 1H, Ar–*H*), 7.76–7.80 (m, 2H, Ar–*H*), 7.95–7.96 (m, 1H, Ar–*H*) 10.88 (s, 1H, Ar-N*H*); ^13^C-NMR (125 MHz, DMSO-*d*_*6*_) *δ* 31.15, 112.31, 119.72, 122.05, 125.71, 126.10, 126.70, 128.01, 128.66, 131.00, 131.77, 132.77, 133.47, 139.98, 158.72, 159.97, 207.03. Anal. calcd. for C_17_H_11_F_3_N_4_O (344.29): C, 61.26; H, 4.23; N, 16.12; found: C, 61.13; H, 4.35; N, 16.29.

#### *N*-(2-Fluorophenyl)-5-(2-(trifluoromethyl)benzyl)-1,3,4-oxadiazol-2-amine (4c)

Color: light green; yield: 72%, M.P: 118–122 °C; IR *υ*_max_ (cm^−1^): 3288 (N–H), 1604 (C=N); ^1^H-NMR (DMSO- *d*^6^) *δ* ppm; 4.35 (s, 2H, C*H*_2_), 7.06–7.09 (m, 1H, Ar–*H*), 7.18 (m, 2H, Ar–*H*), 7.56 (d, 2H, *J* = 7.6 Hz Ar–*H*), 7.71 (t, 1H, *J* = 8.0 Hz Ar–*H*), 7.78 (d, 1H, *J* = 8.0 Hz Ar–*H*), 8.01 (t, 1H, *J* = 8.0 Hz Ar–*H*), 10.22 (s, 1H, Ar-N*H*); ^13^C-NMR (125 MHz, DMSO-*d*_*6*_) *δ* 31.17, 100.50, 115.69, 116.30, 121.25, 123.98, 124.82, 126.47, 128.60, 132.69, 140.78, 149.83, 158.64, 160.85, 207.07. Anal. calcd. for C_16_H_11_F_4_N_3_O (337.27): C, 56.98; H, 3.29; N, 12.46; found: C, 56.79; H, 3.55; N, 12.38.

#### *N*-Phenyl-5-(pyridin-4-yl)-1,3,4-oxadiazol-2-amine (4d)^27^

Color: light brown; yield: 80%, M.P: 180–183 °C; IR *υ*_max_ (cm^−1^): 3262 (N–H), 1590 (C=N); ^1^H-NMR (500 MHz, DMSO-*d*_*6*_) *δ* 1.44–1.53 (m, 1H, C*H*_3_), 7.51 (d, 1H, *J* = 8.0 Hz, Ar–*H*), 7.61 (t, 1H, *J* = 8.0 Hz, Ar–*H*), 7.88 (dd, 1H, *J* = 1.2, 7.6 Hz Ar–*H*), 7.95 (d, 2H, *J* = 8.4 Hz, Ar–*H*) 8.04 (m, 1H, Ar–*H*), 8.10 (d, 2H, *J* = 8.0 Hz, Ar–*H*), 11.27 (s, 1H, Ar–*H*); ^13^C-NMR (125 MHz, DMSO-*d*_*6*_) *δ* 117.75, 119.75, 122.71, 129.64, 131.38, 138.79, 151.26, 156.66, 161.03. Anal. calcd. for C_13_H_10_N_4_O (238.24): C, 65.54; H, 4.23; N, 23.52; found: C, 65.42; H, 4.09; N, 23.39.

#### *N*-(2,4-Dimethylphenyl)-5-(3-fluorophenyl)-1,3,4-oxadiazol-2-amine (4e)

Color: light brown; yield: 73%, M.P: 171–174 °C; IR *υ*_max_ (cm^−1^): 3262 (N–H), 1588 (C=N); ^1^H-NMR (500 MHz, DMSO-*d*_*6*_) *δ* 2.26 (s, 6H, C*H*_3_), 2.30 (s, 3H, Ar–*H*), 7.03 (d, 2H, *J* = 8.0 Hz, Ar–*H*), 7.48 (t, 1H, *J* = 8.0 Hz, Ar–*H*), 7.52–7.55 (m, 1 Hz, Ar–*H*), 7.58–7.60 (m, 2H, Ar–*H*), 9.59 (s, 1H, Ar–*H*), 11.27 (s, 1H, Ar–*H*); ^13^C-NMR (125 MHz, DMSO-*d*_*6*_) *δ* 14.68, 18.27, 20.83, 112.02, 112.27, 121.78, 122.07, 127.42, 129.88, 131.66, 133.63, 134.43, 157.47, 159.91, 162.33, 206.08. Anal. calcd. for C_16_H_14_FN_3_O (283.31): C, 67.83; H, 4.91; N, 14.83; found: C, 67.71; H, 5.05; N, 14.99.

#### 5-(3,5-Difluorobenzyl)-*N*-(2-fluorophenyl)-1,3,4-oxadiazol-2-amine (4f)

Color: off-white; yield: 71%, M.P: 108–112 °C; IR *υ*_max_ (cm^−1^): 3370 (N–H),1655 (C=N); ^1^H-NMR (500 MHz, DMSO-*d*_*6*_) *δ* 4.25 (s, 2H, C*H*_3_), 7.50–7.13 (m, 3H, Ar–*H*), 7.17–7.28 (m, 3H, Ar–*H*), 8.03 (ddd, 1H, *J* = 1.2, 8.4 Hz, Ar–*H*), 10.22 (s, 1H, Ar–*H*), 8.04 (m, 1H, Ar-N*H*); ^13^C-NMR (125 MHz, DMSO-*d*_*6*_) *δ* 31.16, 103.26, 112.66, 112.91, 115.84, 116.02, 120.92, 123.91, 125.18, 139.42, 150.96, 154.01, 158.81, 160.79, 207.08. Anal. calcd. for C_15_H_10_F_3_N_3_O (305.25): C, 59.02; H, 3.30; N, 13.77; found: C, 58.89; H, 3.55; N, 13.59.

#### *N*-(2,4-Difluorophenyl)-5-(3,5-difluorobenzyl)-1,3,4-oxadiazol-2-amine (4g)

Color: light brown; yield: 82%, M.P: 115–118 °C; IR *υ*_max_ (cm^−1^): 3376 (N–H), 1650 (C=N); ^1^H-NMR (500 MHz, DMSO-*d*_*6*_) *δ* 4.29 (s, 2H, C*H*_3_), 7.08–7.21 (m, 4H, Ar–*H*), 7.30–7.36 (m, 1H, Ar–*H*), 7.98–8.04 (m, 1H, Ar–*H*), 10.20 (s, 1H, Ar–*H*); ^13^C-NMR (125 MHz, DMSO-*d*_*6*_) *δ* 31.16, 103.26, 103.52, 104.72, 111.61, 112.83, 112.90, 139.49, 158.83, 160.93, 161.69, 164.14, 207.01. Anal. calcd. for C_15_H_9_F_4_N_3_O (323.25): C, 55.73; H, 2.81; N, 13.00; found: C, 55.56; H, 2.55; N, 13.19.

#### 5-(2-Fluoro-5-methylphenyl)-*N*-(2-fluorophenyl)-1,3,4-oxadiazol-2-amine (4h)

Color: off-white; yield: 75%, M.P: 178–180 °C; IR *υ*_max_ (cm^−1^): 3368 (N–H), 1628 (C=N); ^1^H-NMR (500 MHz, DMSO-*d*_*6*_) *δ* 2.37 (s, 3H, CH_3_), 7.90–7.17 (m, 1H, Ar–H), 7.23–7.27 (m, 1H, Ar–H), 7.29–7.36 (m, 2H, Ar–H), 7.41–7.45 (m, 1H, Ar–H), 8.06 (dd, 1H, *J* = 6.8 Hz, Ar–H); ^13^C-NMR (125 MHz, DMSO-*d*_*6*_) *δ* 20.52, 11.98, 112.10, 115.98, 116.17, 117.34, 121.39, 124.37, 125.27, 126.89, 129.17, 134.94, 156.29, 158.80, 160.87. Anal. calcd. for C_15_H_11_F_2_N_3_O (287.26): C, 62.72; H, 3.86; N, 14.63; found: C, 62.58; H, 3.65; N, 14.79.

#### *N*-(2-Fluorophenyl)-5-(4-(trifluoromethyl)phenyl)-1,3,4-oxadiazol-2-amine (4i)

Color: off-white; yield: 74%, M.P: 220–222 °C; IR *υ*_max_ (cm^−1^): 3355 (N–H), 1633 (C=N); ^1^H-NMR (500 MHz, DMSO-*d*_*6*_) *δ* 7.10–7.15 (m, 1H, C*H*_3_), 7.24 (t, 1H, *J* = 8.0 Hz, Ar–*H*), 7.28–7.33 (m, 1H, Ar–*H*), 7.95 (d, 2H, *J* = 8.4 Hz, Ar–*H*), 8.08–8.13 (m, 3H, Ar–*H*), 10.62 (s, 1H, Ar–*H*); ^13^C-NMR (125 MHz, DMSO-*d*_*6*_) *δ* 116.20, 121.43, 124.49, 125.28, 125.32, 126.64, 126.74, 126.89, 127.98, 128.90, 157.77, 161.13. Anal. calcd. for C_15_H_9_F_4_N_3_O (323.25): C, 55.73; H, 2.88; N, 13.00; found: C, 55.64; H, 2.81; N, 13.13.

#### 3-((5-(Pyridin-4-yl)-1,3,4-oxadiazol-2-yl)amino)benzonitrile (4j)

Color: off-white; yield: 75%, M.P: 210–213 °C; IR *υ*_max_ (cm^−1^): 3372 (N–H),1620 (C=N); ^1^H-NMR (500 MHz, DMSO-*d*_*6*_) *δ* 7.50 (d, 1H, *J* = 7.6 Hz), 7.61 (t, 1H, *J* = 7.6 Hz, Ar–H), 7.83 (d, 2H, *J* = 6.0 Hz, Ar–H), 7.88 (d, 1H, *J* = 7.6, 8.4 Hz, Ar–H), 8.05 (t,1H, *J* = 1.6 Hz), 8.80 (d, 2H, *J* = 4.4 Hz), 11.33 (s, 1H); ^13^C-NMR (125 MHz, DMSO-*d*_*6*_) *δ* 117.42, 119.62, 122.10, 129.55, 131.35, 139.78, 154.35, 156.32, 160.86, 207.03. Anal. calcd. for C_14_H_9_N_5_O (263.25): C, 63.87; H, 3.45; N, 26.60; found: C, 63.77; H, 3.55; N, 26.89.

#### *N*-(2,4-Dimethylphenyl)-5-(pyridin-4-yl)-1,3,4-oxadiazol-2-amine (4k)

Color: light green; yield: 75%, M.P: 243–246 °C; IR *υ*_max_ (cm^−1^): 3274 (N–H), 1576 (C=N); ^1^H-NMR (500 MHz, DMSO-*d*_*6*_) *δ* 2.09 (s, 3H, C*H*_3_), 7.51 (d, 1H, *J* = 8.0 Hz, Ar–*H*), 7.61 (t, 1H, *J* = 8.0 Hz, Ar–*H*), 7.88 (dd, 1H, *J* = 1.2 Hz 7.6 Hz Ar–*H*), 7.95 (d, 2H, *J* = 8.4 Hz, Ar–*H*) 8.04 (m, 1H, Ar–*H*) 8.10 (d, 2H, *J* = 8.0 Hz, Ar–*H*), 11.27 (s, 1H, Ar–*H*); ^13^C-NMR (125 MHz, DMSO-*d*_*6*_) *δ* 18.31, 20.77, 119.54, 121.82, 122.61, 127.48, 130.26, 131.11, 134.16, 151.01, 151.25, 161.97, 164.74. Anal. calcd. for C_15_H_14_N_4_O (266.30): C, 67.65; H, 5.30; N, 21.04; found: C, 67.44; H, 5.13; N, 21.01.

#### *N*-(4-Bromophenyl)-5-(4-(trifluoromethyl)phenyl)-1,3,4-oxadiazol-2-amine (4l)

Color: off-white; yield: 74%, M.P: 258–262 °C; IR *υ*_max_ (cm^−1^): 3298 (N–H), 1588 (C=N); ^1^H-NMR (500 MHz, DMSO-*d*_*6*_) *δ* 7.43 (dd, 2H, *J* = 4.8 Hz), 7.66 (dd, 2H, *J* = 2.0, 7.2 Hz, Ar–*H*), 7.96 (d, 2H, *J* = 8.4 Hz, Ar–*H*), 8.10 (d, 2H, *J* = 8.0 Hz, Ar–*H*), 10.98 (s, 1H, Ar-N*H*); ^13^C-NMR (125 MHz, DMSO-*d*_*6*_) *δ* 119.25, 125.69, 126.21, 126.81, 126.86, 127.98, 129.50, 131.25, 137.90, 157.40, 160.57. Anal. calcd. for C_15_H_9_BrF_3_N_3_O (384.15): C, 46.90; H, 2.36; N, 10.96; found: C, 46.82; H, 2.45; N, 10.89.

#### *N*-(4-Isopropylphenyl)-5-(4-(trifluoromethyl)phenyl)-1,3,4-oxadiazol-2-amine (4m)

Color: off-white; yield: 73%, M.P: 219–222 °C; IR *υ*_max_ (cm^−1^): 3322 (N–H), 1580 (C=N); ^1^H-NMR (500 MHz, DMSO-*d*_*6*_) *δ* 1.19 (d, 6H, *J* = 6.4 Hz, C*H*_3_), 2.83–2.88 (m, 1H, Ar–*H*), 7.24 (d, 2H, *J* = 8.4 Hz, Ar–*H*), 7.53 (dd, 2H, *J* = 1.6, 8.0 Hz, Ar–*H*), 7.94 (d, 2H, *J* = 8.4 Hz, Ar–*H*) 8.08 (d, 2H, *J* = 8.4 Hz, Ar–*H*),10.69 (s, 1H, Ar–*H*); ^13^C-NMR (125 MHz, DMSO-*d*_*6*_) *δ* 24.47, 33.26, 117.81, 123.04, 125.74, 126.68, 126.85, 127.35, 128.03, 136.66, 142.72, 157.13, 160.95. Anal. calcd. for C_18_H_16_F_3_N_3_O (347.33): C, 62.24; H, 4.64; N, 12.10; found: C, 62.02; H, 4.42; N, 12.01.

#### 5-Phenyl-*N*-(*o*-tolyl)-1,3,4-oxadiazol-2-amine (4n)^28^

Color: off-white; yield: 83%, M.P: 154–157 °C; IR *υ*_max_ (cm^−1^): 3262 (N–H), 1575 (C=N); ^1^H-NMR (500 MHz, DMSO-*d*_*6*_) *δ* 2.31 (s, 3H, C*H*_3_), 7.05 (t, 1H, *J* = 5.6 Hz, Ar–*H*), 7.22–7.24 (m, 2H, Ar–*H*), 7.56–7.58 (m, 3H, Ar–*H*), 7.77 (d, 1H, *J* = 8.4 Hz, Ar–*H*), 7.87–7.89 (m, 2H, Ar–*H*), 9.65 (s, 1H, Ar–*H*); ^13^C-NMR (125 MHz, DMSO-*d*_*6*_) *δ* 18.62, 123.42, 123.84, 126.10, 126.58, 127.54, 128.71, 129.04, 129.25, 131.33, 142.65, 164.36, 169.32. Anal. calcd. for C_15_H_13_N_3_O (251.28): C, 71.70; H, 5.21; N, 16.72; found: C, 71.51; H, 5.09; N, 16.89.

#### 5-(3-Fluoro-2-methylphenyl)-*N*-phenyl-1,3,4-oxadiazol-2-amine (4o)

Color: off-white; yield: 81%, M.P: 147–151 °C; IR *υ*_max_ (cm^−1^): 3363 (N–H), 1594 (C=N); ^1^H-NMR (500 MHz, DMSO-*d*_*6*_) *δ* 2.41 (s, 3H, C*H*_3_), 7.07–7.23 (m, 1H, Ar–*H*), 7.23–7.29 (m, 2H, Ar–*H*), 7.46–7.48 (m, 3H, Ar–*H*), 7.87 (d, 1H, *J* = 4.8 Hz, Ar–*H*) 7.95 (d, 2H, *J* = 5.7 Hz, Ar–*H*), 10.42 (s, 1H, Ar–*H*); ^13^C-NMR (125 MHz, DMSO-*d*_*6*_) *δ* 12.85, 115.42, 117.18, 123.00, 123.08, 124.84, 129.91, 138.46, 138.92, 160.00, 164.55, 169.36. Anal. calcd. for C_15_H_12_FN_3_O (269.27): C, 66.91; H, 4.49; N, 15.60; found: C, 66.78; H, 4.55; N, 15.47.

### 4.3 Monoamine oxidase A and B inhibition assay

Newly synthesized compounds were tested on monoamine oxidase A and B (extracted). Freshly prepared enzyme extracts were used in fluorescence-based assays *via* estimating the change in fluorescence intensity in white colored 96 well plate with an excitation at 544 nm and an emission at 590 nm. To determine the enzyme activity of MAO and MAO-B. Clorgyline 6 mM and Deprenyl 10 mM were employed as standard inhibitors of MAO-A and MAO-B, respectively. Reaction mixture was composed of 100 μL of total assay volume having 30 μL buffer (pH 7.4), 10 μL test compounds, 10 μL of extracted enzyme (MAO-A: 13 μg mL^−1^, MAO-B: 15 μg mL^−1^), 30 μL of amplex red and 20 μL of substrate (p-tyramine). Initially reaction mixture having buffer + test compound + enzyme was incubated for 15 minutes for MAO-A And MAO-B. After Incubation the amplex red reagent was added followed by addition of substrate p-tyramine. The change in the fluorescence was determined by using fluorescence plate reader: FLUOstar® Omega (BMG Labtech GmbH, orten berg Germany). Those compounds exhibited greater than 50% inhibition of either the MAO-A or MAO-B activity, were further subjected for the estimation of IC_50_ values. All experiments were performed in triplicate. IC_50_ values were calculated by non-linear curve fitting program PRISM 5.0 (GraphPad, San Diego, California, USA) where log inhibitor *vs.* response curve was generated. The details of the enzyme inhibition assay for AChE and BChE are inserted in the ESI.†

### 4.4 Docking studies protocol

Molecular docking studies were performed by using FlexX utility of BioSolveIT's LeadIT. The purpose was to analyze the binding behavior of identified inhibitors of MAO-A/MAO-B, AChE/BChE at the active site of enzyme. Crystal structures of MAO-A (2Z5X), MAO-B (2V5Z) and AChE (4BDT) were downloaded from protein data bank (www.rcsb.org).^2^^27–29^ For the revalidation of docking protocol, initially the co-crystalized ligand was redocked at active site and its RMSD value was compared. The scoring and ranking of conformational poses were carried out *via* adopting enthalpy entropy hybrid approach of the FlexX utility. The highest scoring poses were further subjected to HYDE assessment in order to assess their binding affinities.^30,31^

## Conflicts of interest

The authors declare that they have no significant conflict of interest.

## Supplementary Material

RA-013-D3RA01953E-s001

RA-013-D3RA01953E-s002
