# Supplementary material for: Therapeutic potential of 1,3,4-oxadiazoles as potential lead compounds for the treatment of Alzheimer's disease
Source: RSC Adv. 2023 Jun 9;13(26):17526–35. doi: 10.1039/d3ra01953e (PMC10253498; doi:10.1039/d3ra01953e)

Electronic Supplementary Material (ESI) for RSC Advances.
This journal is © The Royal Society of Chemistry 2023

Supplementary Material

Therapeutic Potential of 1,3,4-oxadiazoles as potential lead compounds for the treatment of Alzheimer's disease

Saira Naseem[1Ψ], Ahmed Temirak[2Ψ], Aqeel Imran[3], Saquib Jalil[3], Shamool Fatima[1], Parham Taslimi[4], Jamshed Iqbal[3*], Mussarat Tasleem[1], Muhammad Tahir Nawaz[5] and Zahid Shafiq[*1]

[1]*Institute of Chemical Sciences, Bahauddin Zakariya University, Multan 60800, Pakistan*

[2]*National Research Centre, Chemistry of Natural and Microbial Products Department, Pharmaceutical and Drug Industries Research Institute, Dokki, Cairo P.O. Box 12622, Egypt*

[3]*Centre for Advanced Drug Research, COMSATS University Islamabad, Abbottabad Campus, Abbottabad-22060, Pakistan*

[4]*Department of Biotechnology, Faculty of Science, Bartin University, 74100 Bartin, Turkey*

[5]*Department of Physics, university of Sargodha, Sargodha, Pakistan*

**Corresponding Author*:

Prof. Dr. Zahid Shafiq: zahidshafiq@bzu.edu.pk

Prof. Dr. Jamshed Iqbal: drjamshed@cuiatd.edu.pk

Ψ = Authors contributed equally

Table S1. Experimental crystal structure details of compound **4n**.

**CCDC**	
Chemical formula	$C_{15}H_{13}N_3O$
M_r	251.28
Crystal system, space group	Monoclinic, $P2_1/c$
Temperature (K)	296
a, *b*, *c* (Å)	8.5156 (16), 7.2615 (15), 20.971 (4)
β (°)	93.424 (9)
V (Å^3)	1294.4 (4)
Z	4
Radiation type	Mo $K\alpha$
μ (mm^{-1})	0.08
Crystal size (mm)	0.38 × 0.32 × 0.30
**Data collection**	
Diffractometer	Bruker Kappa APEXII CCD
Absorption correction	Multi-scan (*SADABS*; Bruker, 2005)
No. of measured, independent and observed [$I > 2\sigma(I)$] reflections	11008, 3064, 2374
R_{int}	0.057
$(\sin \theta/\lambda)_{max}$ (Å^{-1})	0.659
**Refinement**	
$R[F^2 > 2\sigma(F^2)]$, $wR(F^2)$, S	0.055, 0.160, 1.05
No. of reflections	3064
No. of parameters	173
H-atom treatment	H-atom parameters constrained
$\Delta\rho_{max}$, $\Delta\rho_{min}$ (e Å^{-3})	0.24, −0.23

Table S2. Hydrogen-bond geometry (Å, º) in compound **4n**.

D*—H···*A	***D*—H**	**H···*A***	***D*···*A***	***D*—H···*A***
N1—H1···N2^{i}	0.86	2.10	2.9373 (19)	163

Symmetry code: (i) $-x+1, -y+1, -z$.

Table S3. Computational toxicological evaluation for Compounds **4a-o**.

Code	Predicted Toxicity class	Predicted LD50	Predicted end points of toxicity
**4a**	4	550mg/kg	Inactive for the Immunotoxicity, mutagenicity and cytotoxicity, carcinogenicity
**4b**	4	800mg/kg	Inactive for the Immunotoxicity, mutagenicity and cytotoxicity, carcinogenicity
**4c**	4	800mg/kg	Inactive for the Immunotoxicity, mutagenicity and cytotoxicity, carcinogenicity
**4d**	4	580mg/kg	Inactive for the Immunotoxicity, mutagenicity and cytotoxicity, carcinogenicity
**4e**	4	550mg/kg	Inactive for the Immunotoxicity, mutagenicity and cytotoxicity, carcinogenicity
**4f**	4	550mg/kg	Inactive for the Immunotoxicity, mutagenicity and cytotoxicity, carcinogenicity
**4g**	4	550mg/kg	Inactive for the Immunotoxicity, mutagenicity and cytotoxicity, carcinogenicity
**4h**	4	550mg/kg	Inactive for the Immunotoxicity, mutagenicity and cytotoxicity, carcinogenicity
**4i**	4	1190mg/kg	Inactive for the Immunotoxicity, mutagenicity and cytotoxicity, carcinogenicity
**4j**	4	580mg/kg	Inactive for the Immunotoxicity, mutagenicity and cytotoxicity, carcinogenicity
**4k**	4	580mg/kg	Inactive for the Immunotoxicity, mutagenicity and cytotoxicity, carcinogenicity
**4l**	4	550mg/Kg	Inactive for the Immunotoxicity, mutagenicity and cytotoxicity, carcinogenicity
**4m**	4	550mg/Kg	Inactive for the Immunotoxicity, mutagenicity and cytotoxicity, carcinogenicity
**4n**	4	550mg/Kg	Inactive for the Immunotoxicity, mutagenicity and cytotoxicity, carcinogenicity
**4o**	4	1190mg/kg	Inactive for the Immunotoxicity, mutagenicity and cytotoxicity, carcinogenicity

H[1] & ^{13}CNMR of Compound 4a

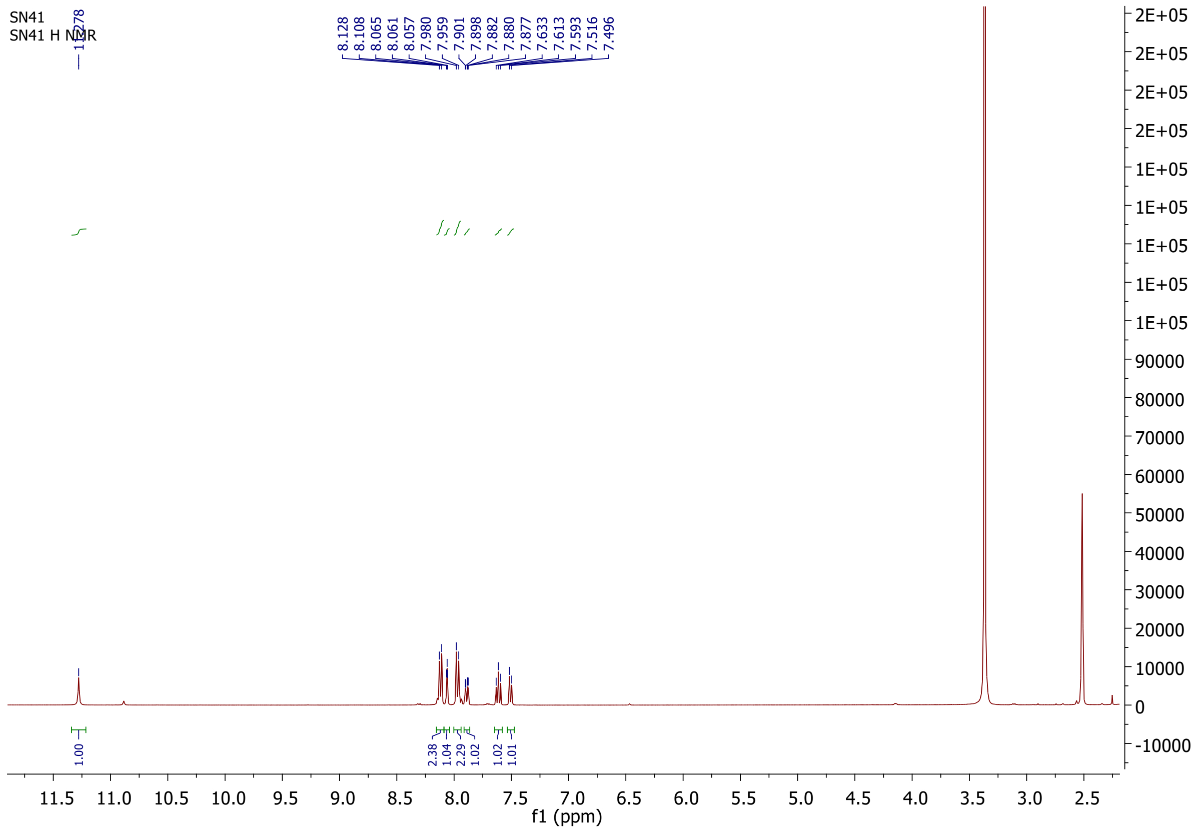

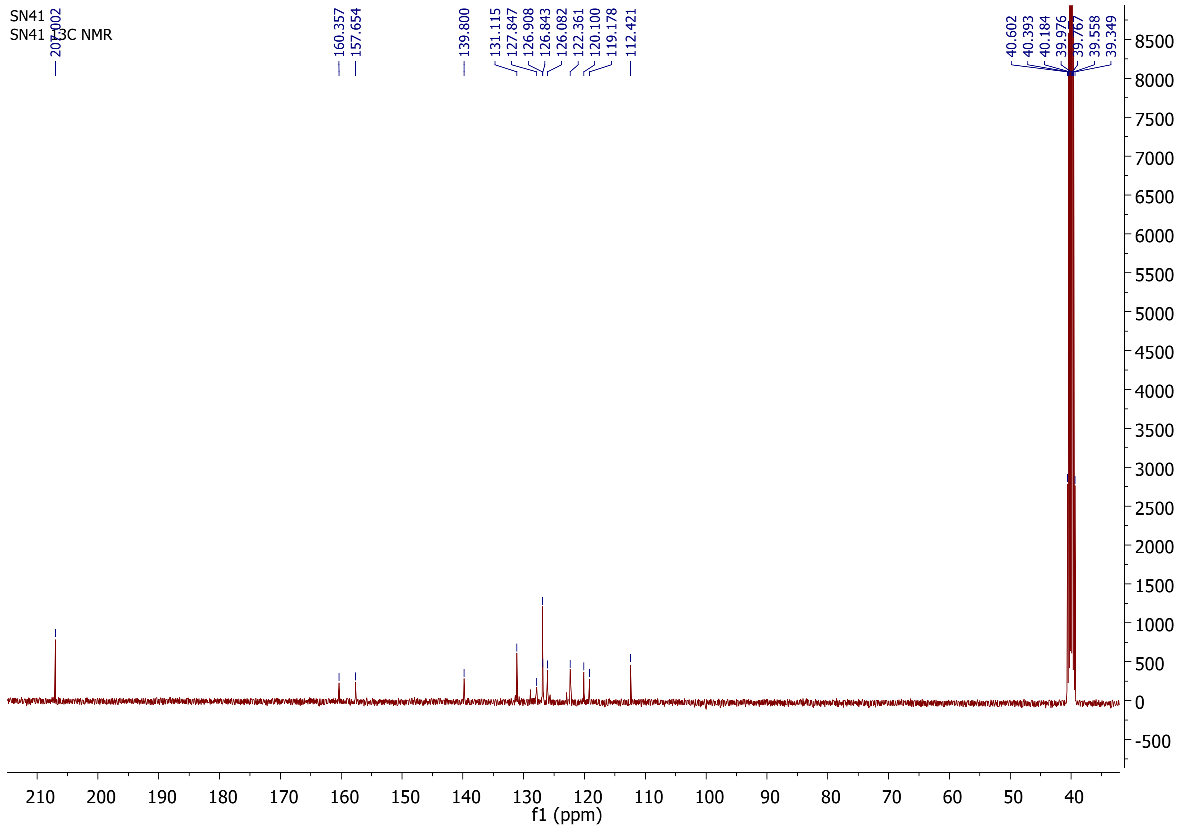

H[1] & ^{13}CNMR of Compound 4b

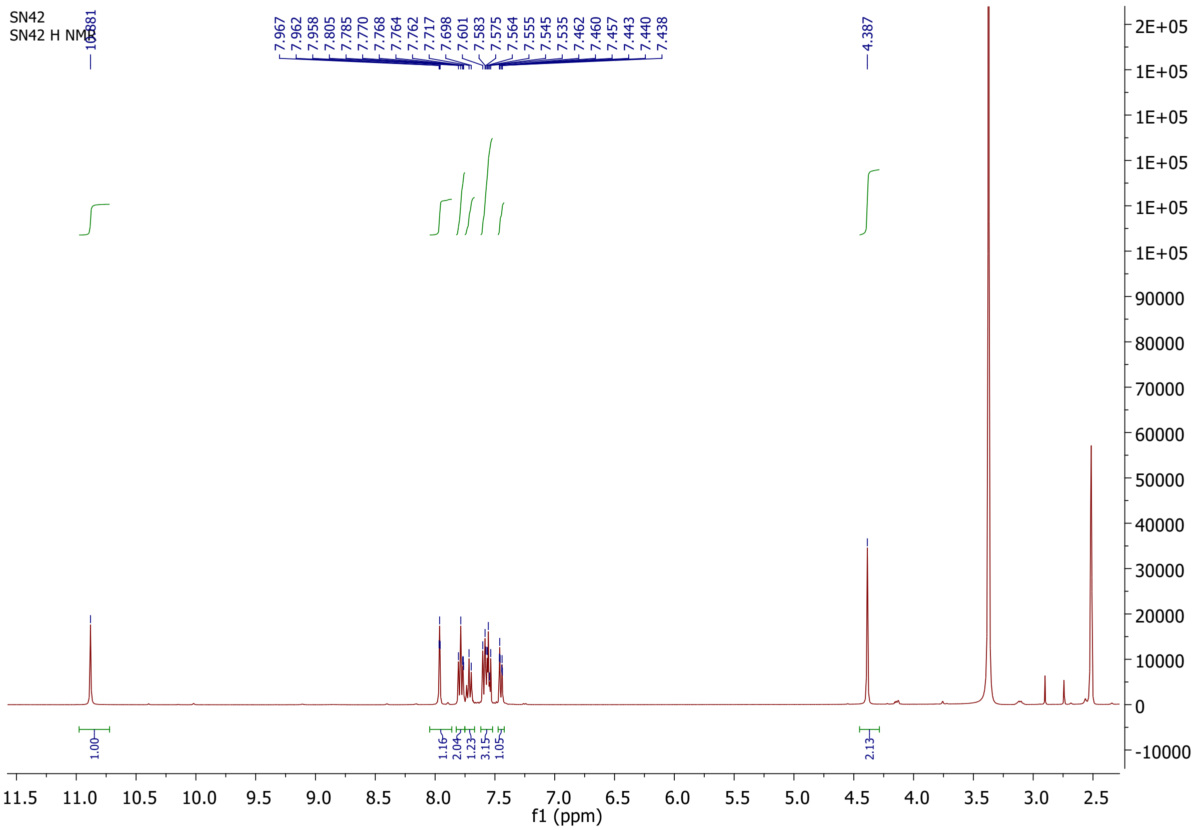

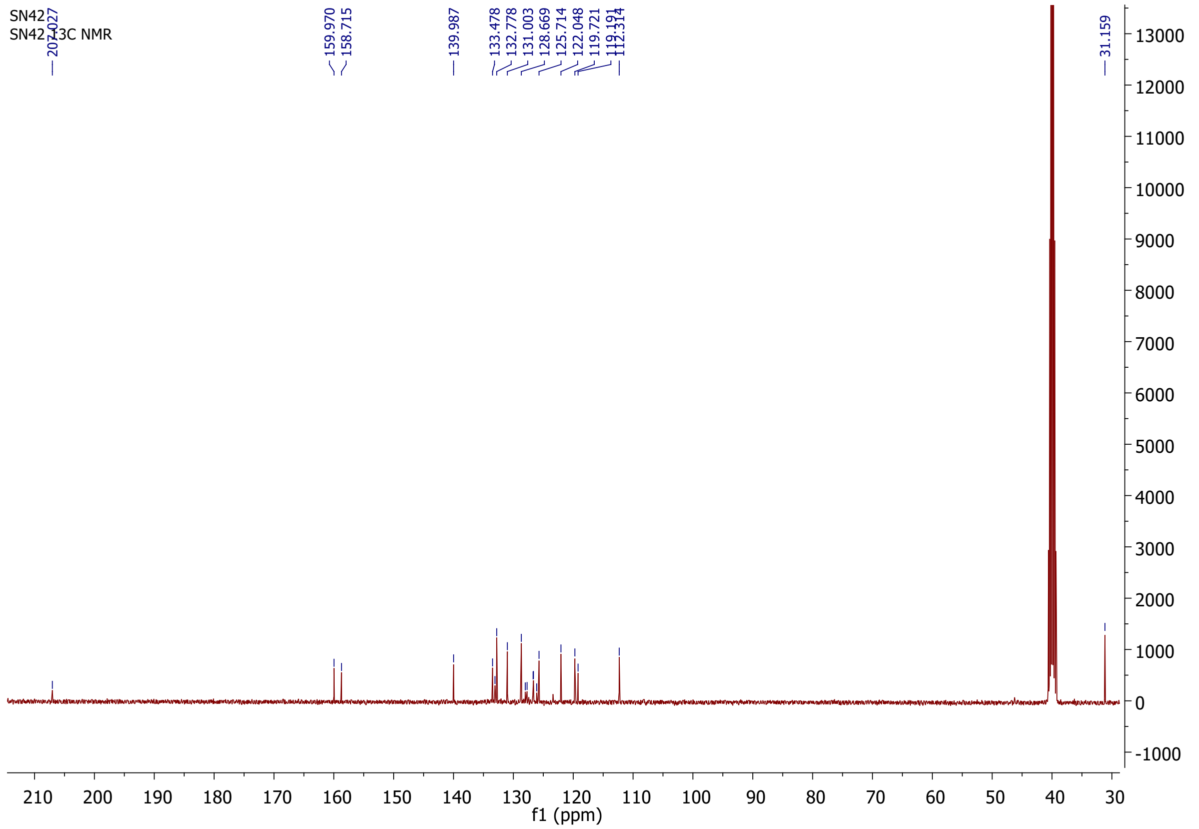

H[1] & ^{13}CNMR of Compound 4c

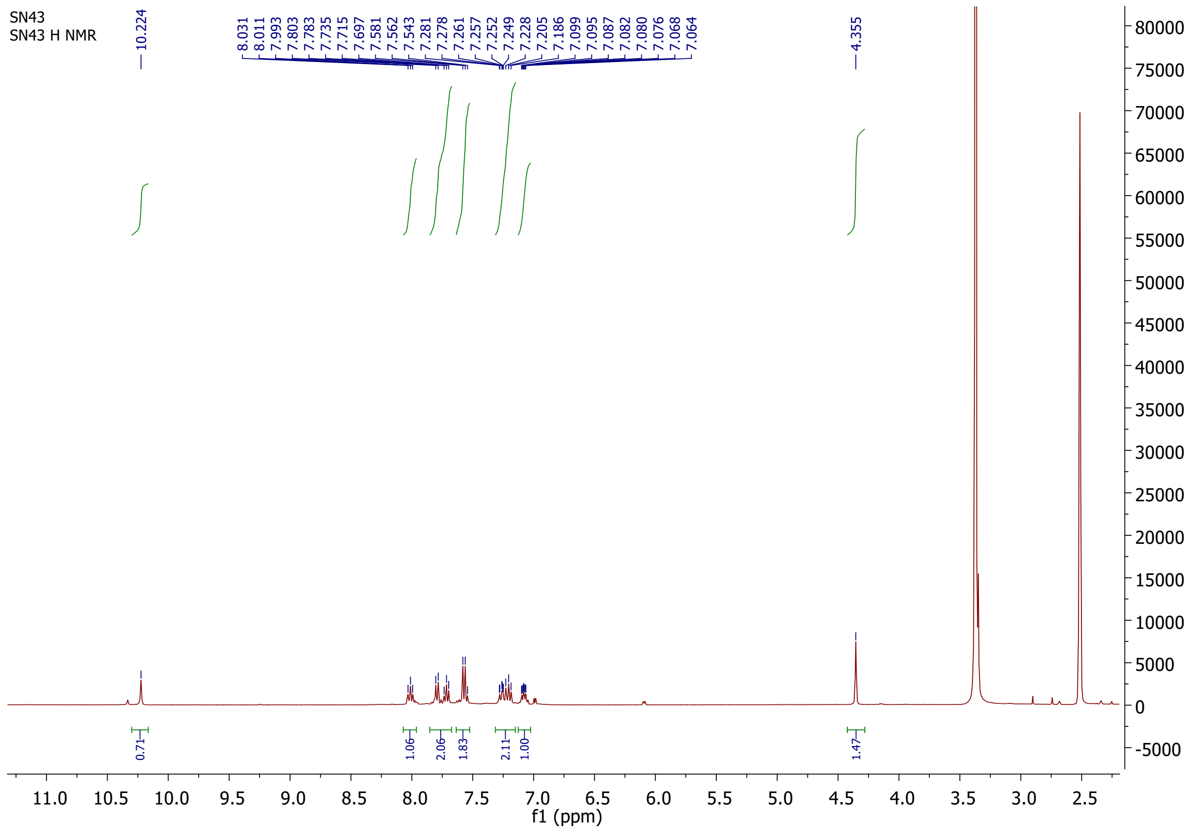

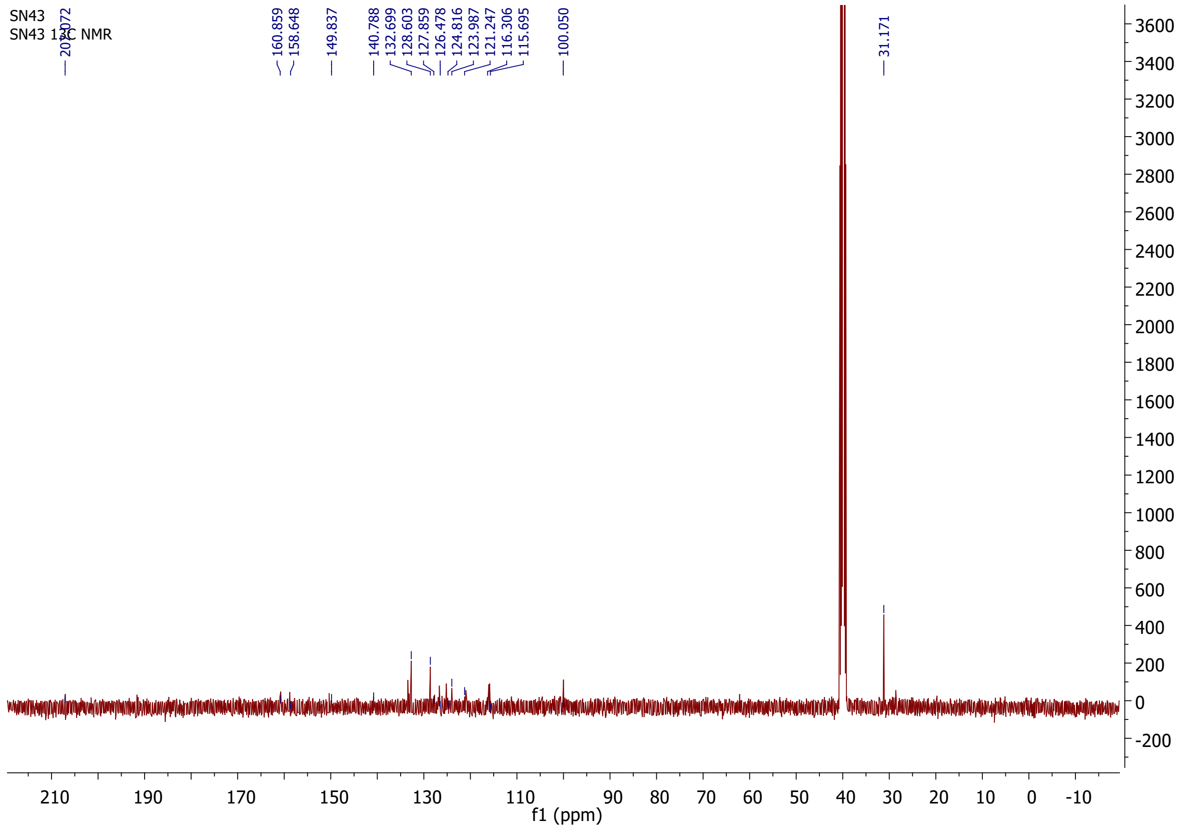

H[1] & ^{13}CNMR of Compound 4d

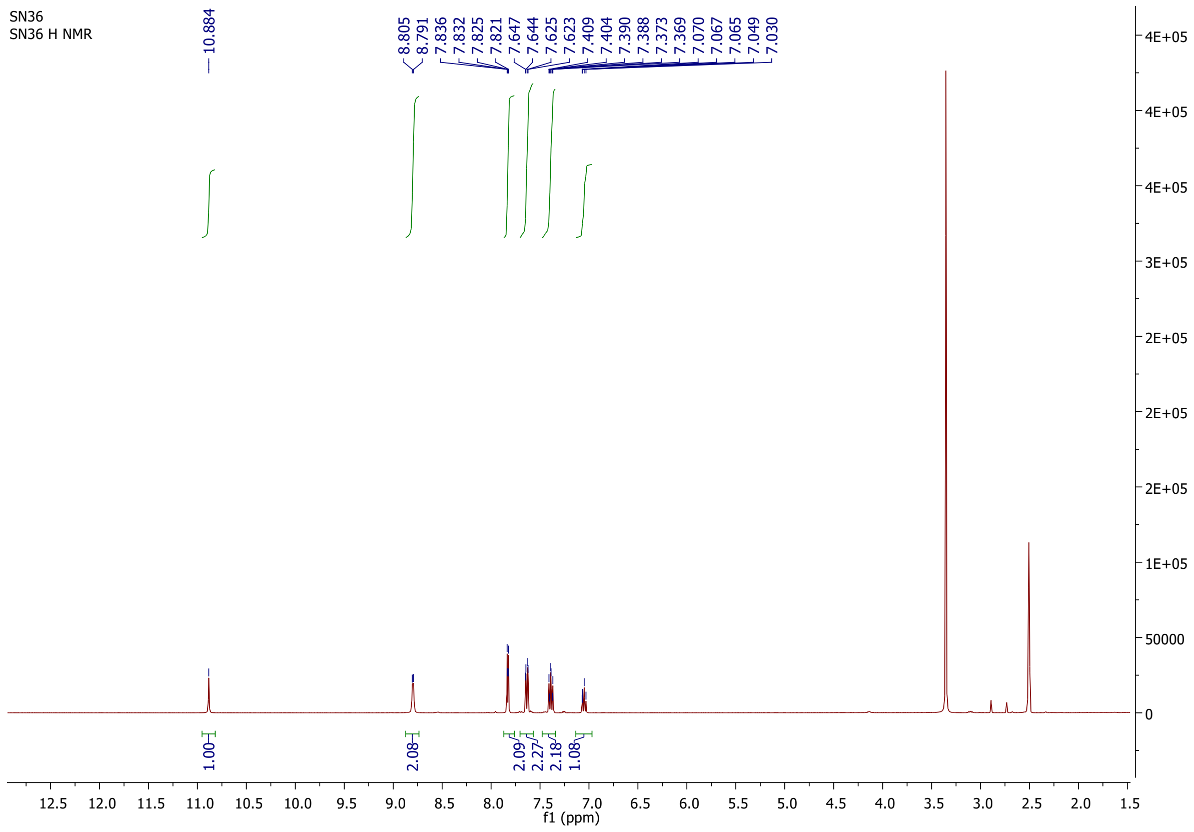

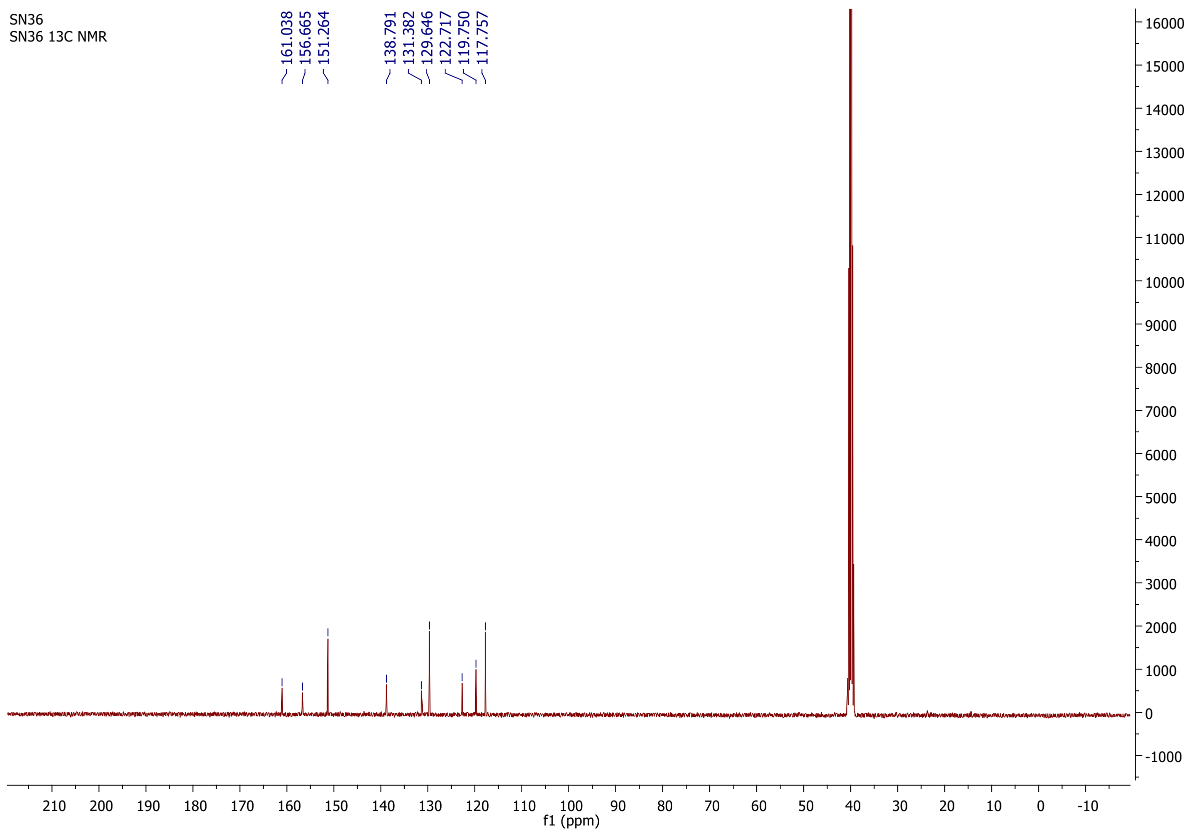

H[1] & ^{13}CNMR of Compound 4e

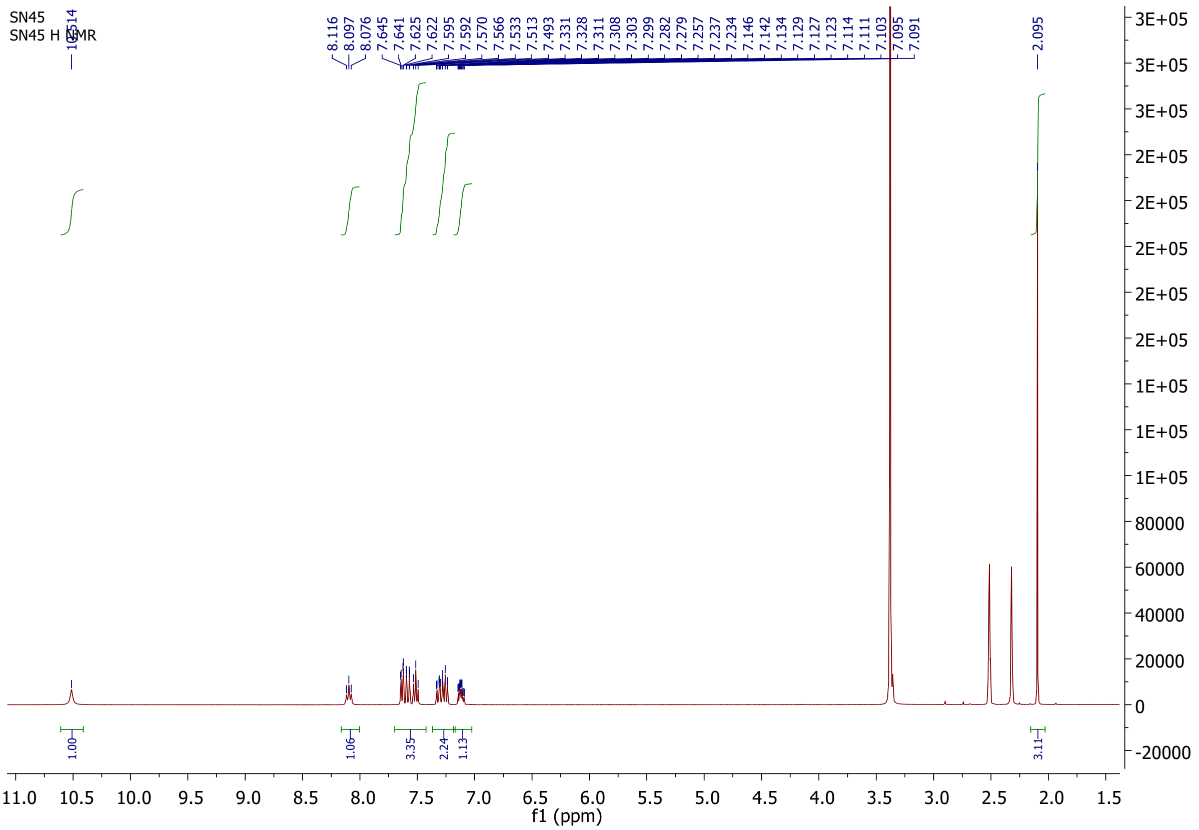

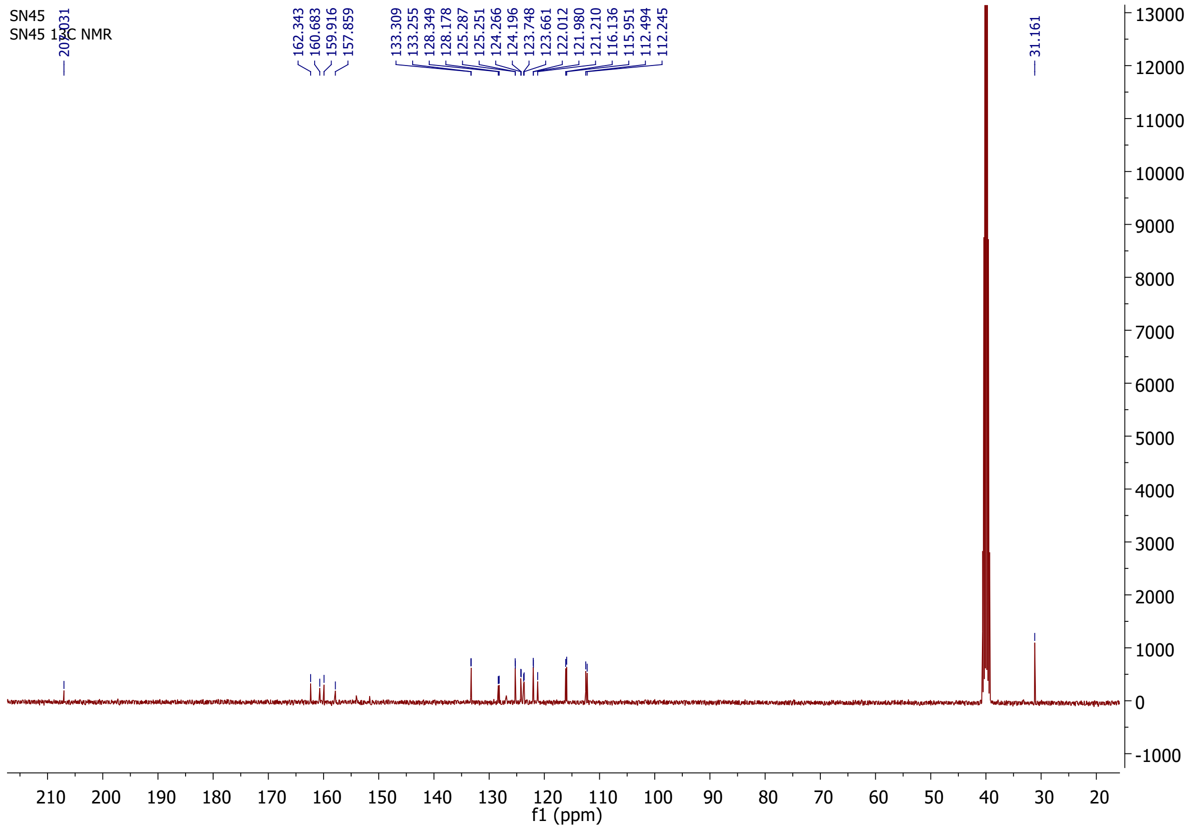

H[1] & ^{13}CNMR of Compound 4f

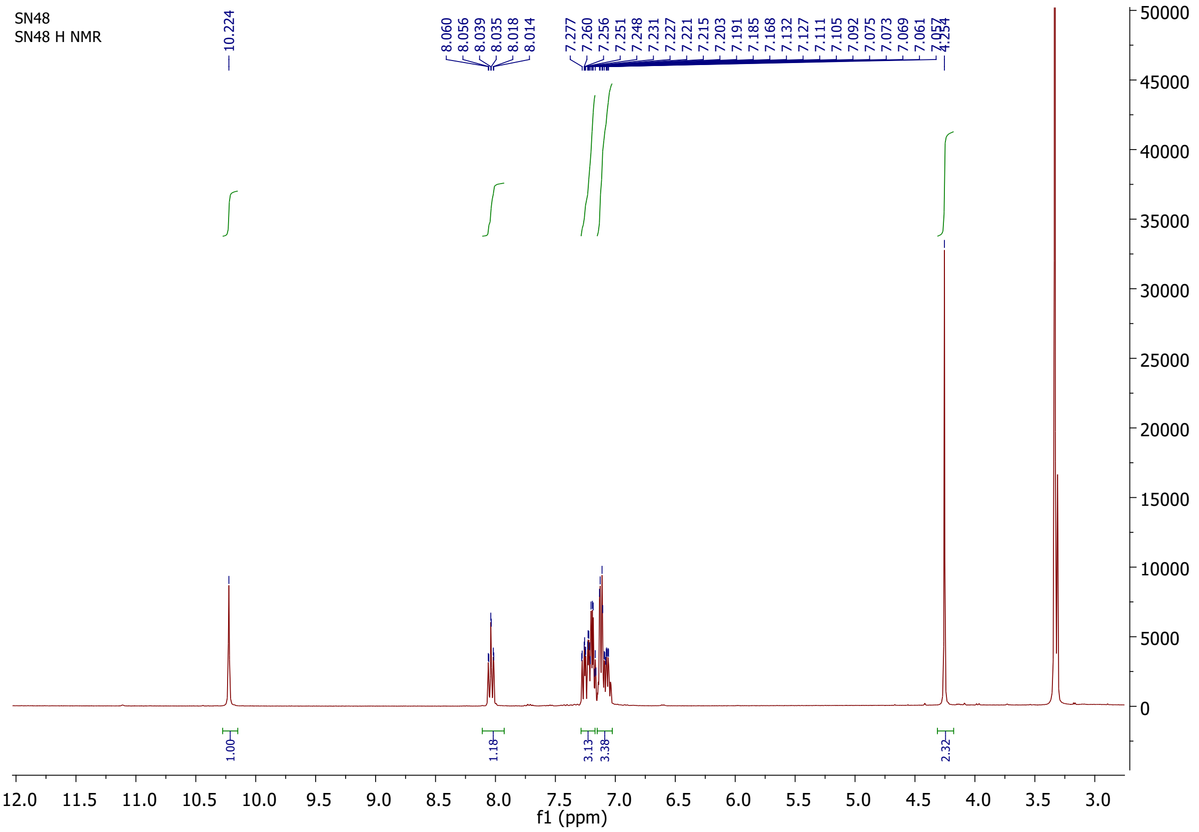

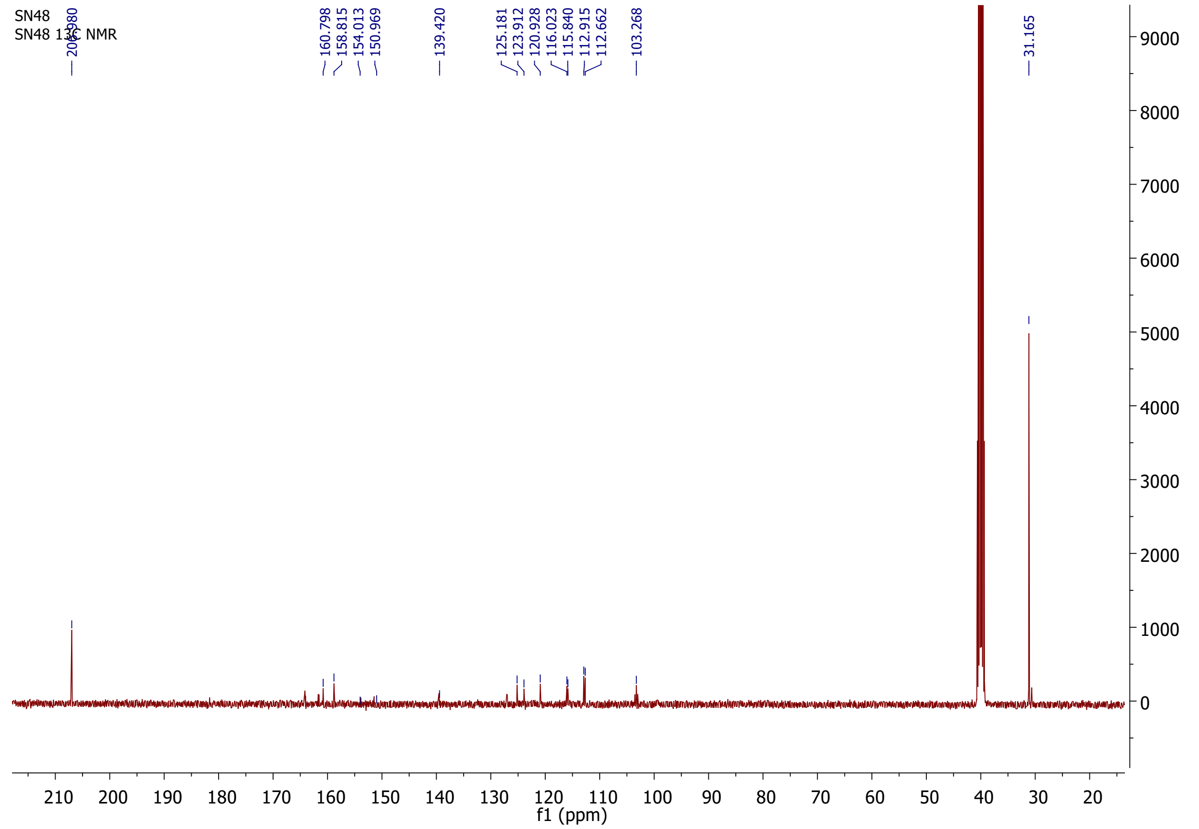

H[1] & ^{13}CNMR of Compound 4g

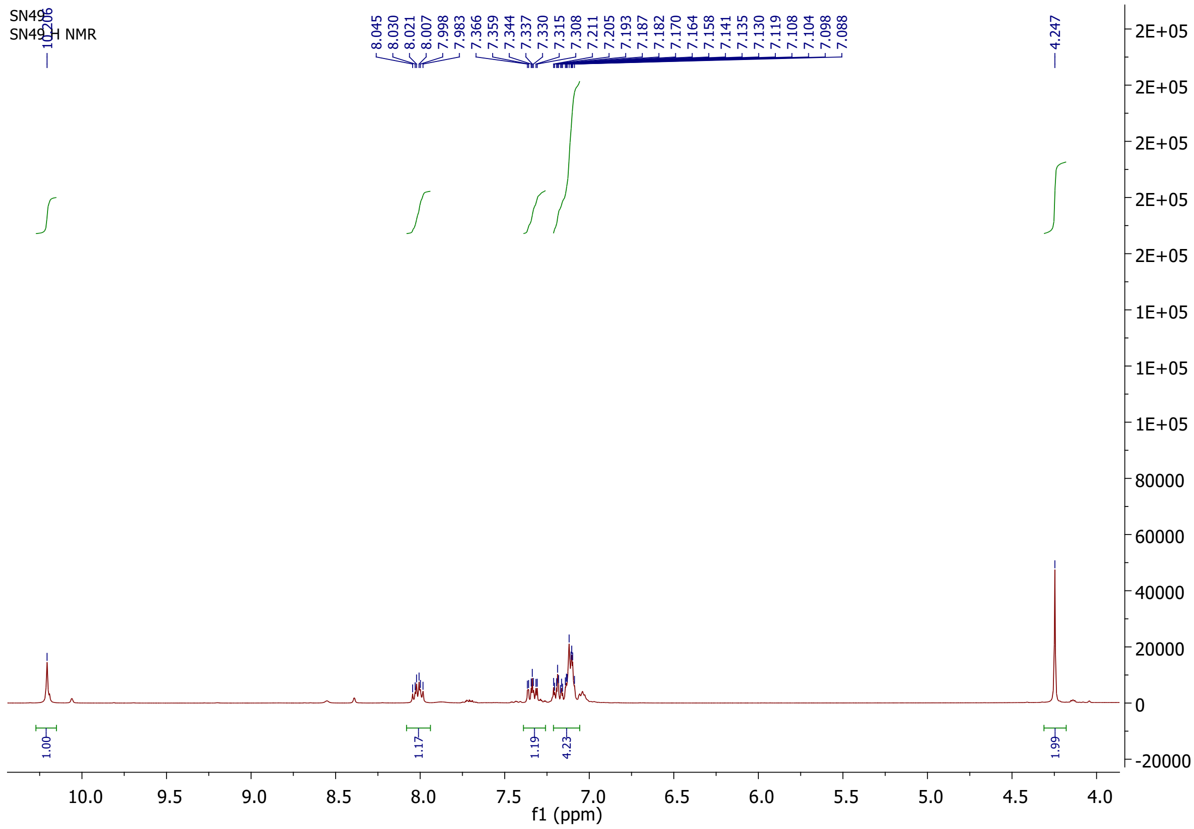

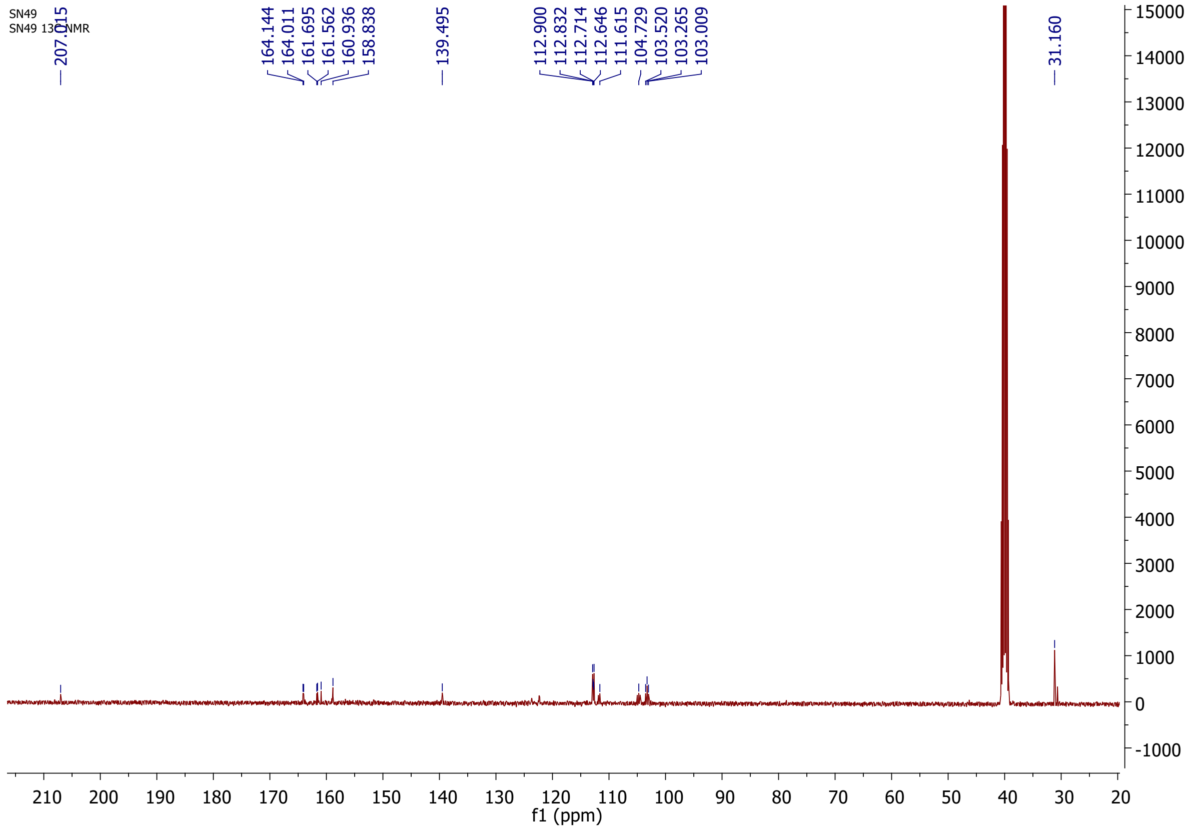

H[1] & ^{13}CNMR of Compound 4h

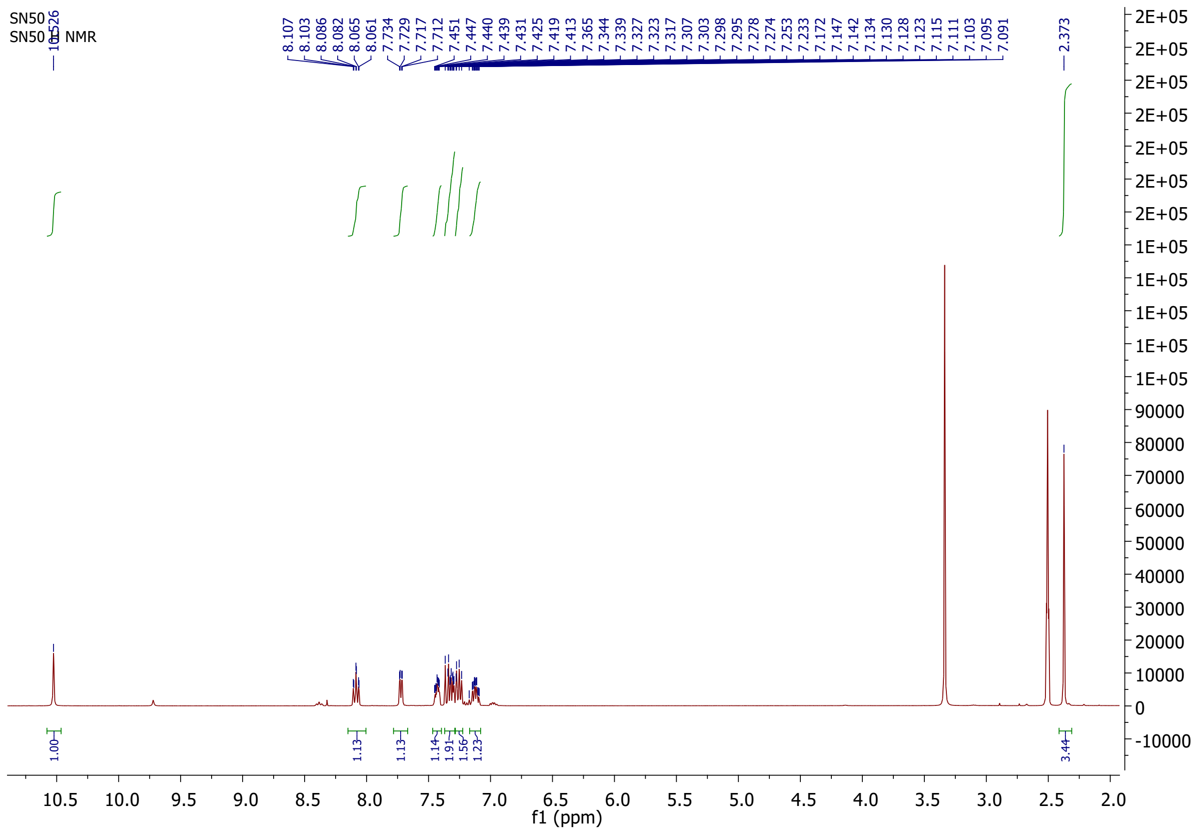

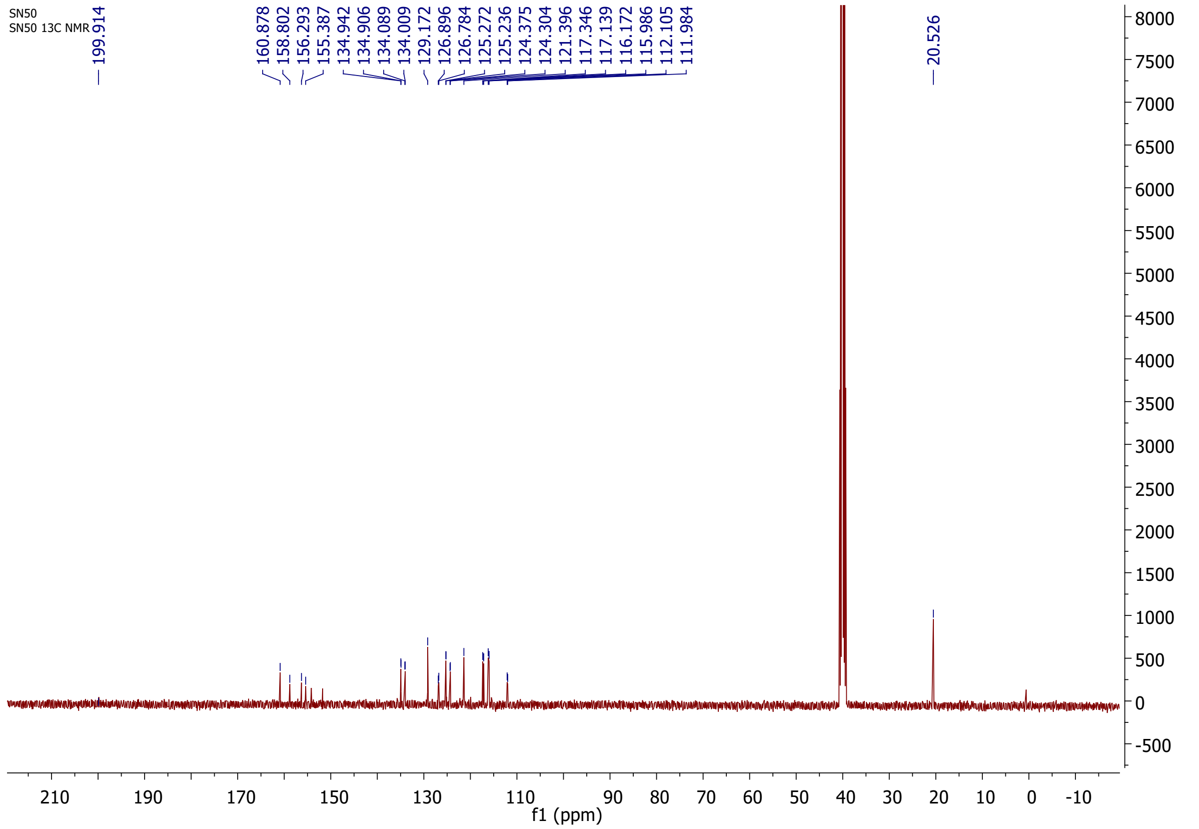

H[1] & ^{13}CNMR of Compound 4i

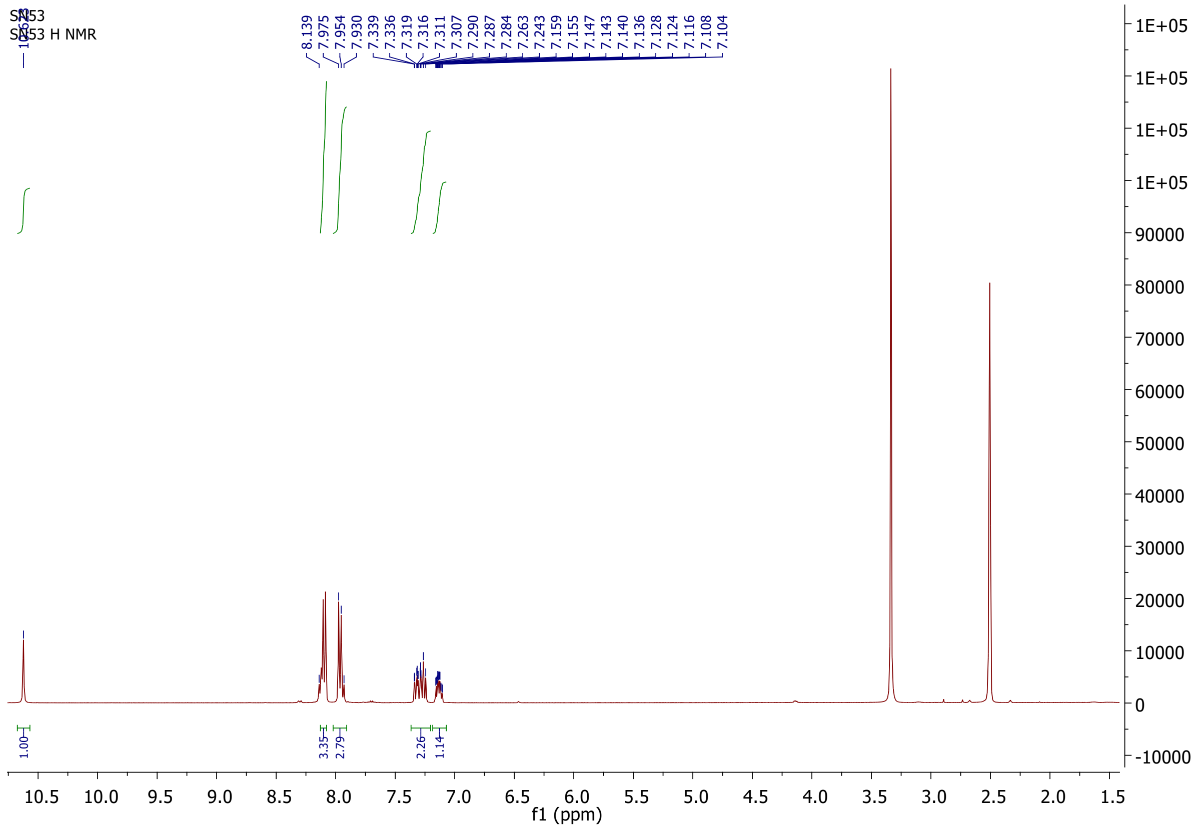

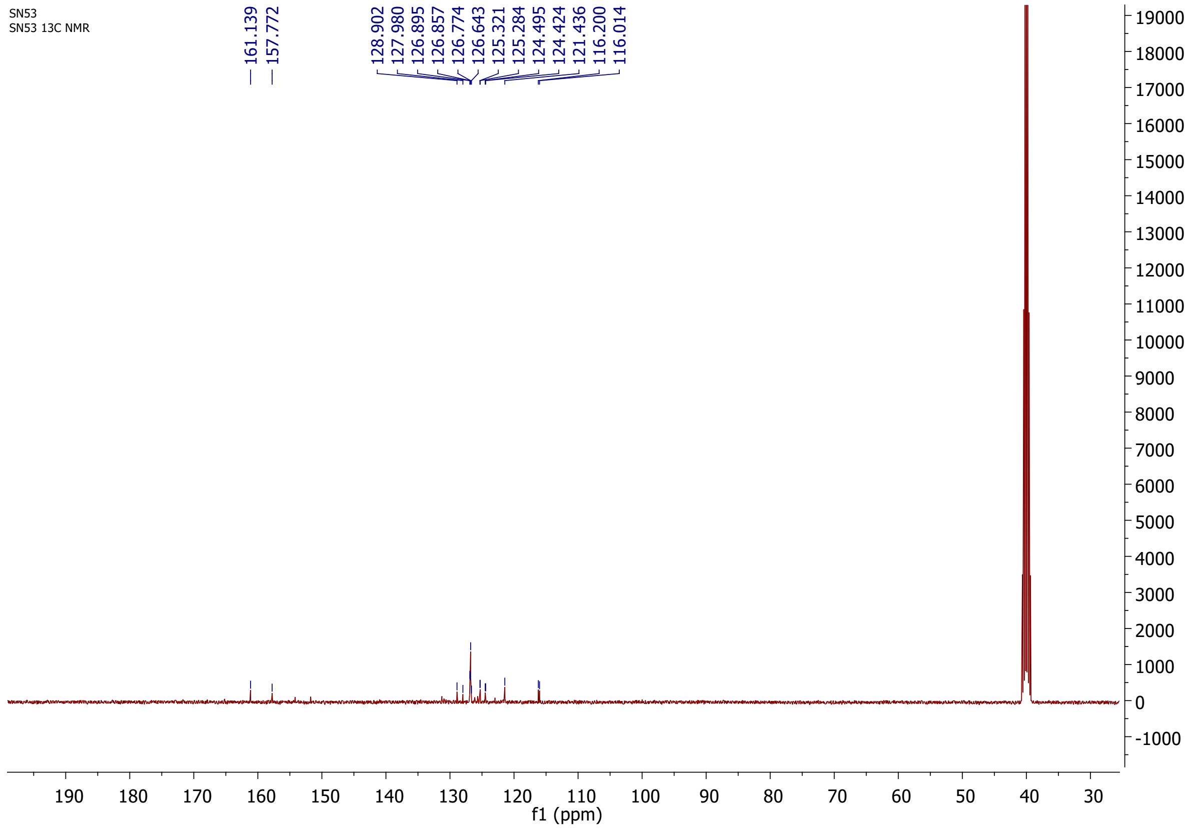

H[1] & ^{13}CNMR of Compound 4j

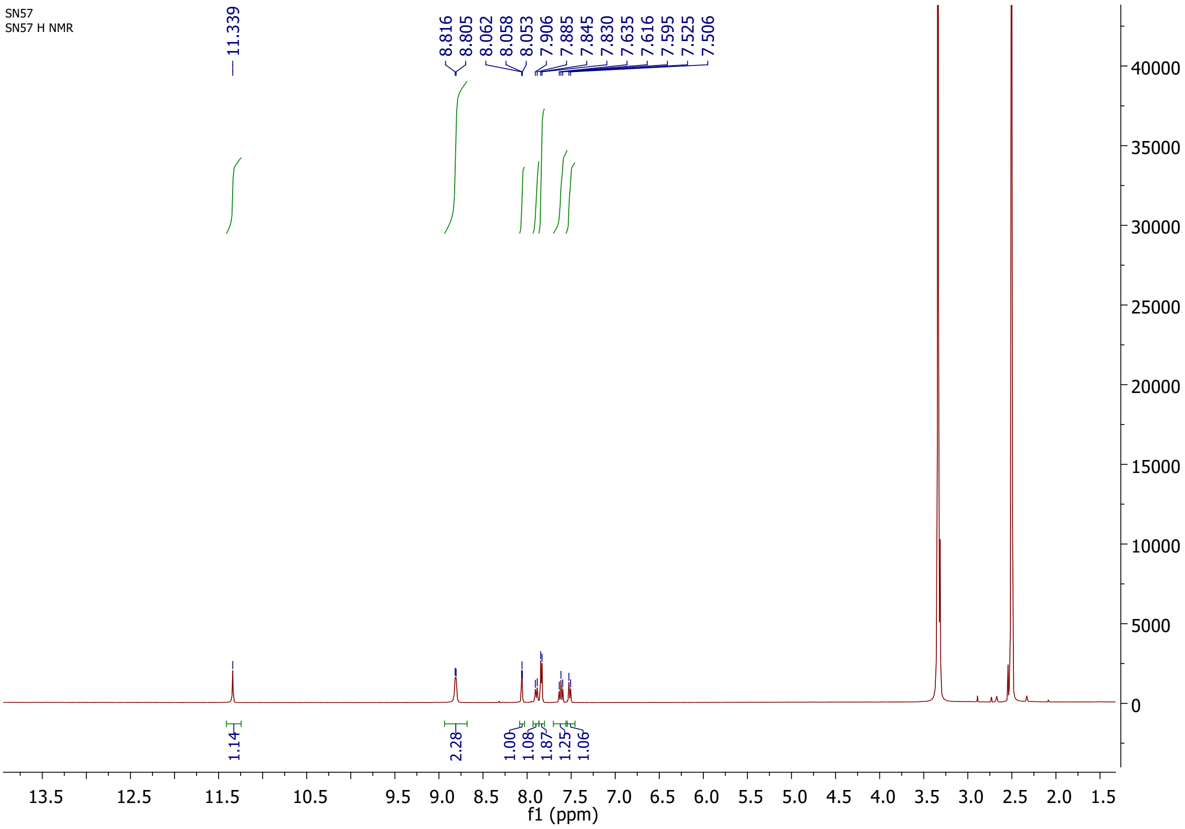
SN57
SN57 H NMR
11.339
8.816
8.805
8.062
8.058
8.053
7.906
7.885
7.845
7.830
7.635
7.616
7.595
7.525
7.506
1.14
2.28
1.00
1.08
1.87
1.25
1.06
f1 (ppm)

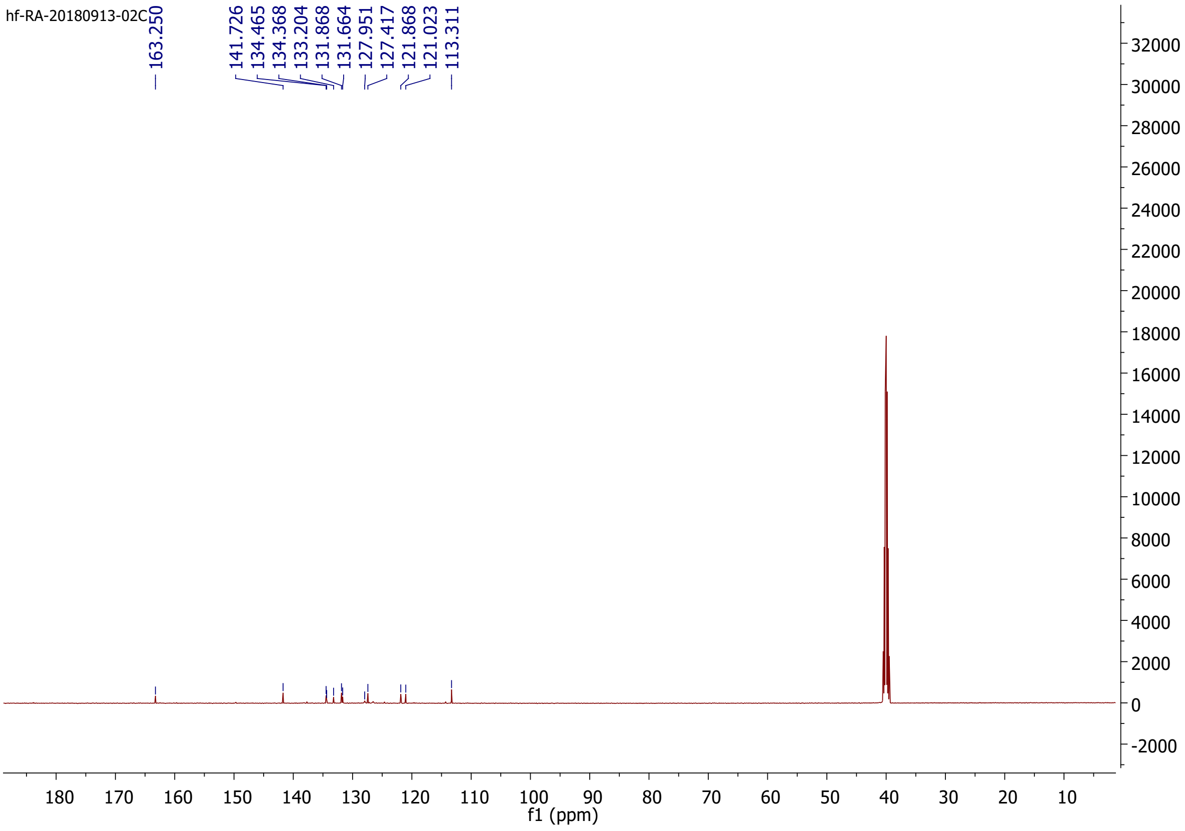
hf-RA-20180913-02C
163.250
141.726
134.465
134.368
133.204
131.868
131.664
127.951
127.417
121.868
121.023
113.311
f1 (ppm)

H[1] & ^{13}CNMR of Compound 4k

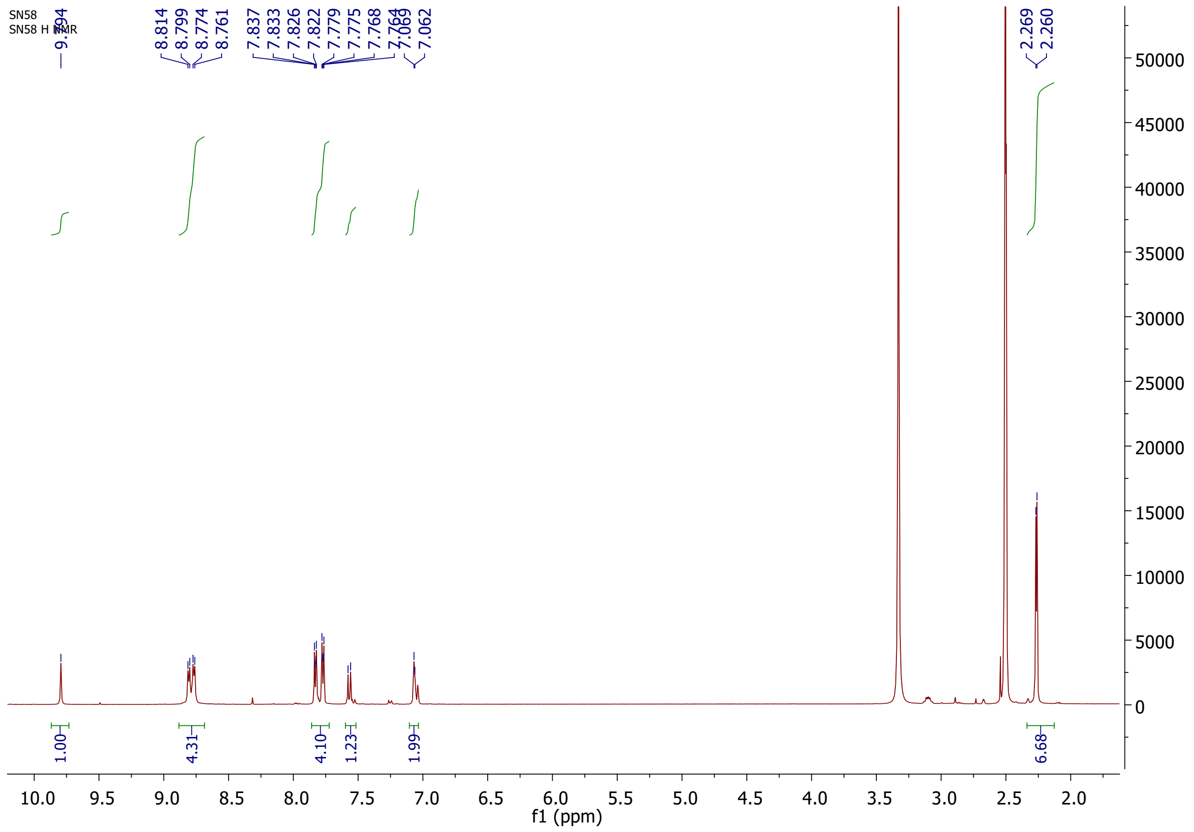

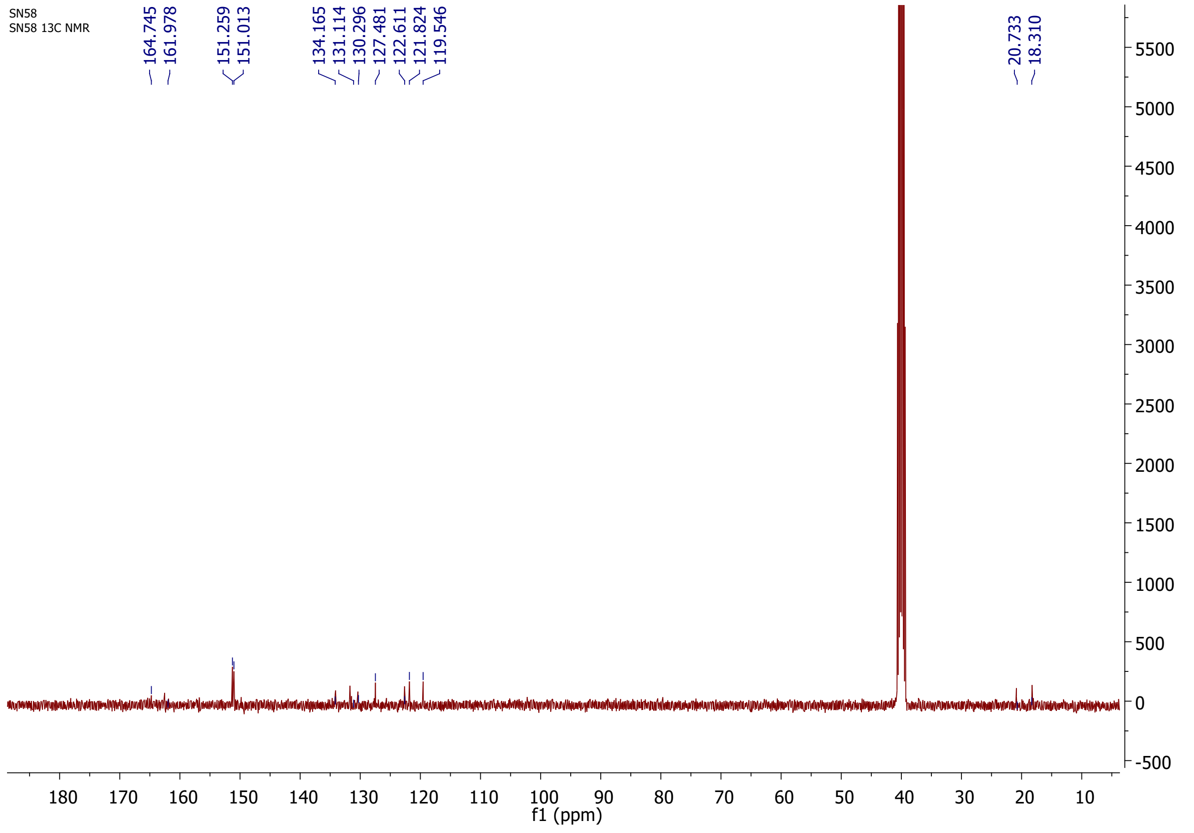

H[1] & ^{13}CNMR of Compound 4l

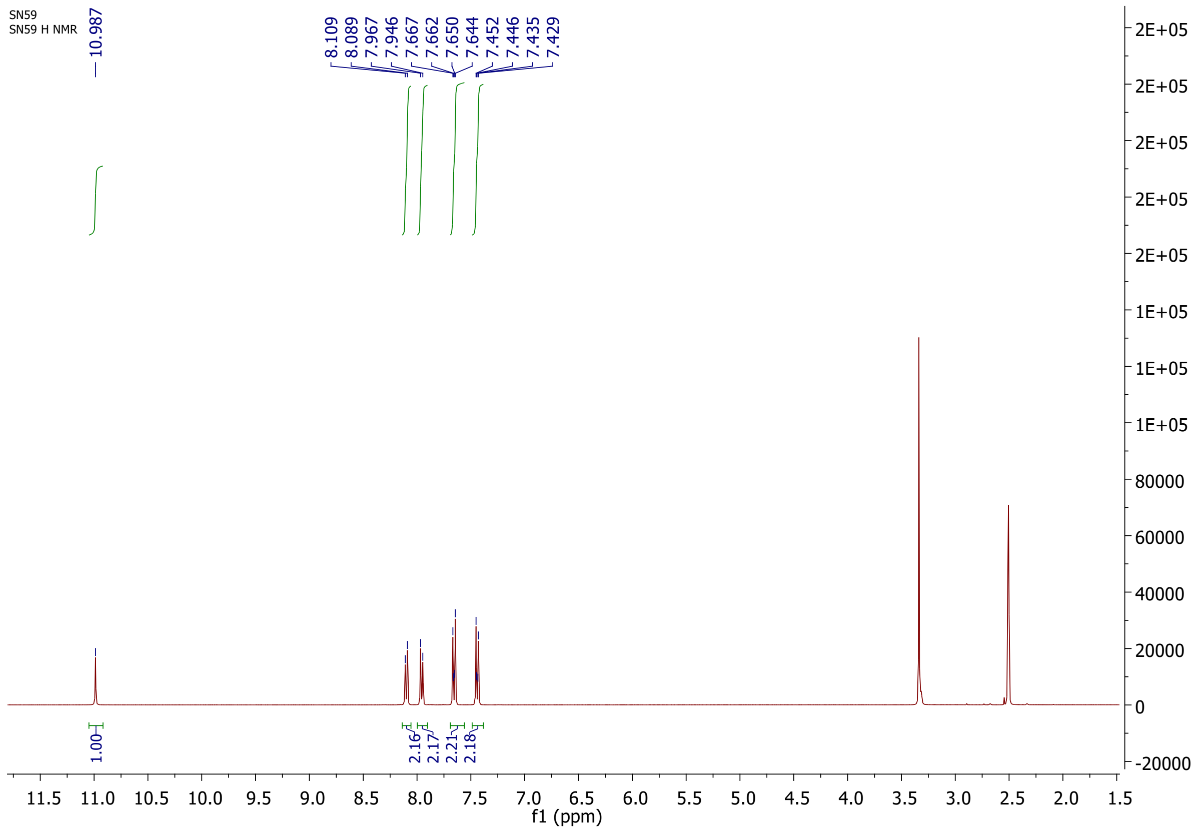

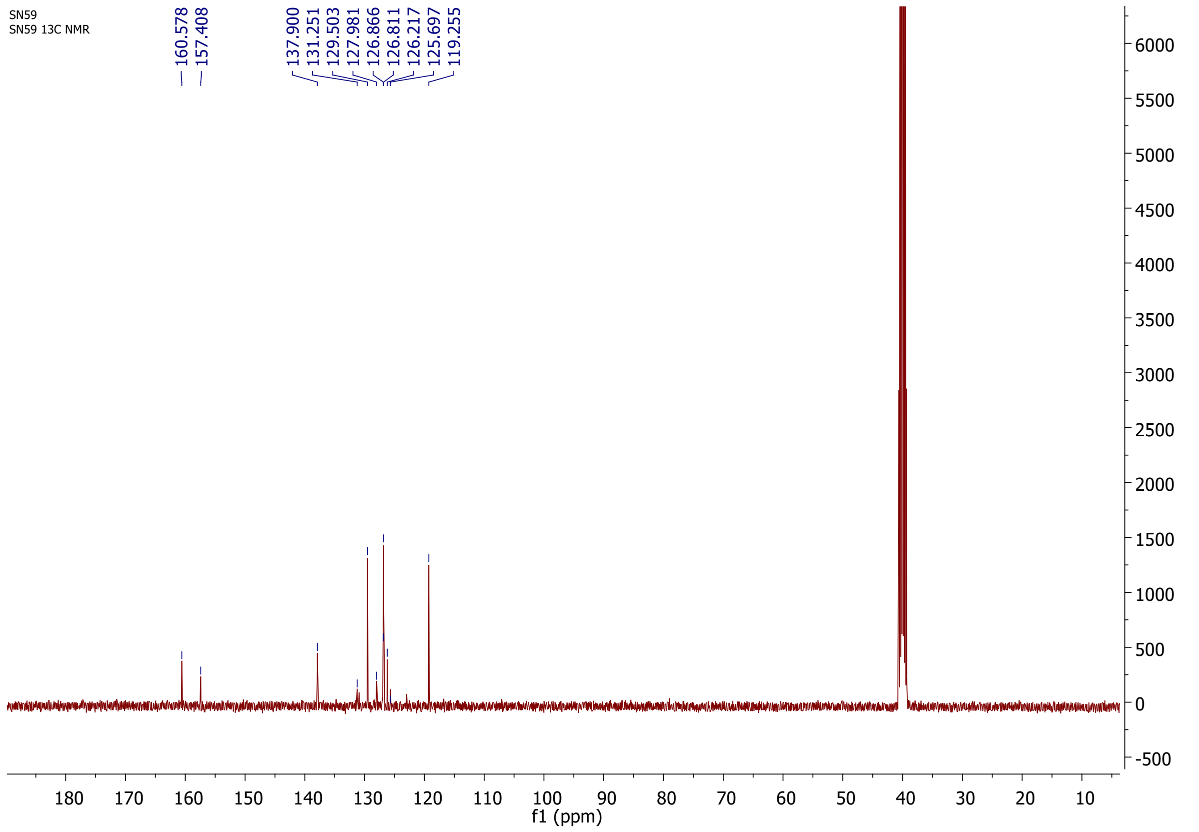

H^1 & ^{13}CNMR of Compound 4m

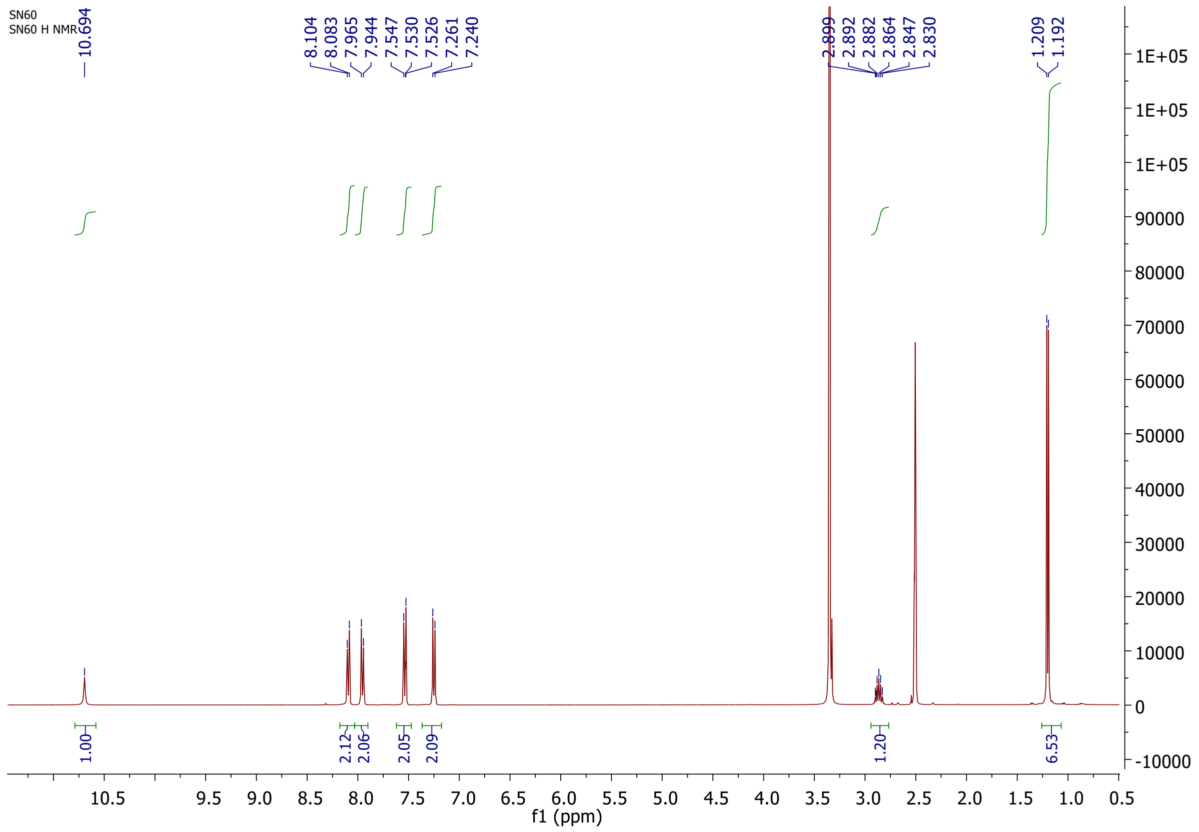

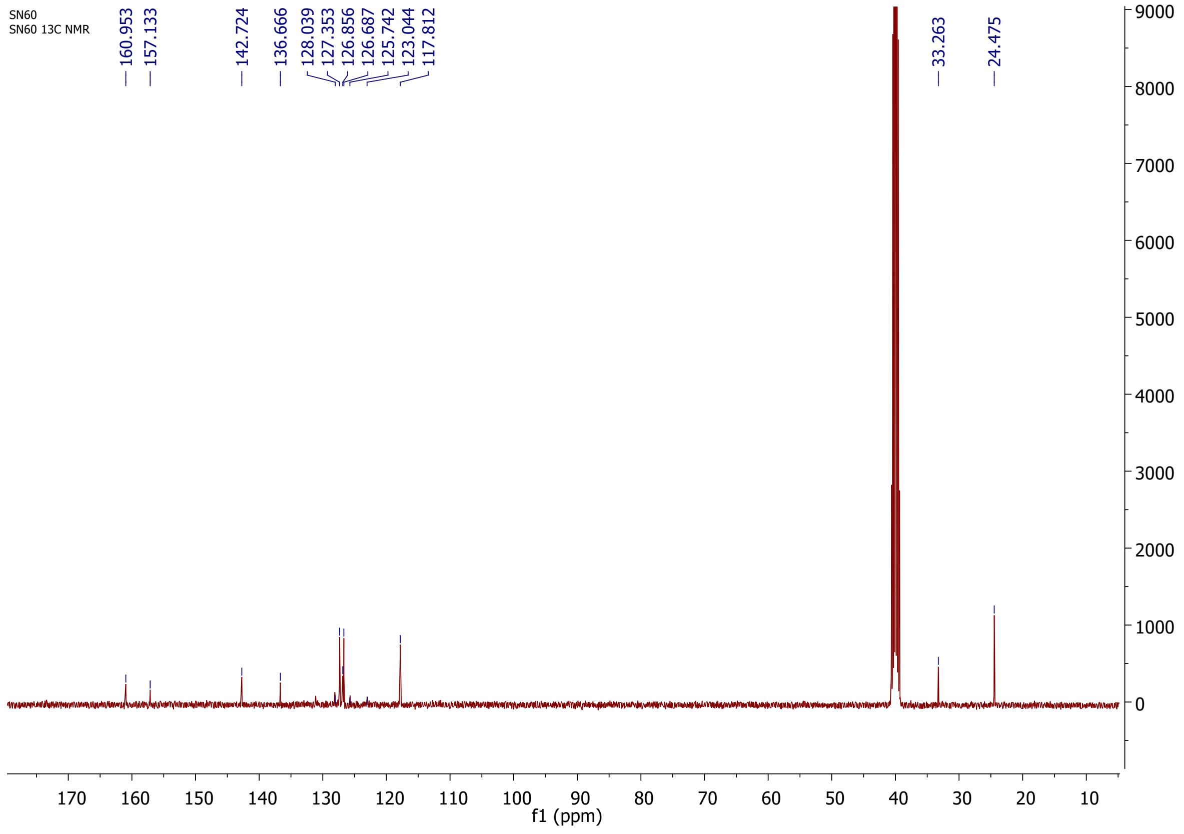

H[1] & ^{13}CNMR of Compound 4n

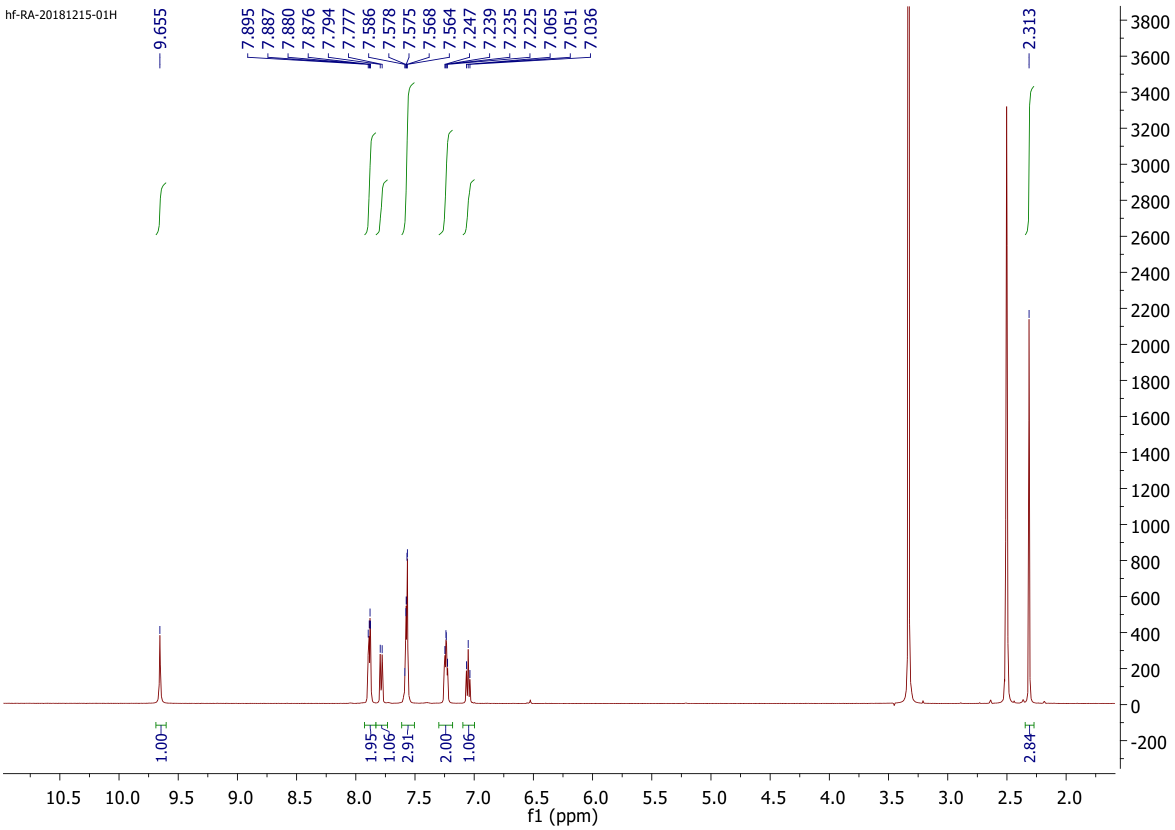

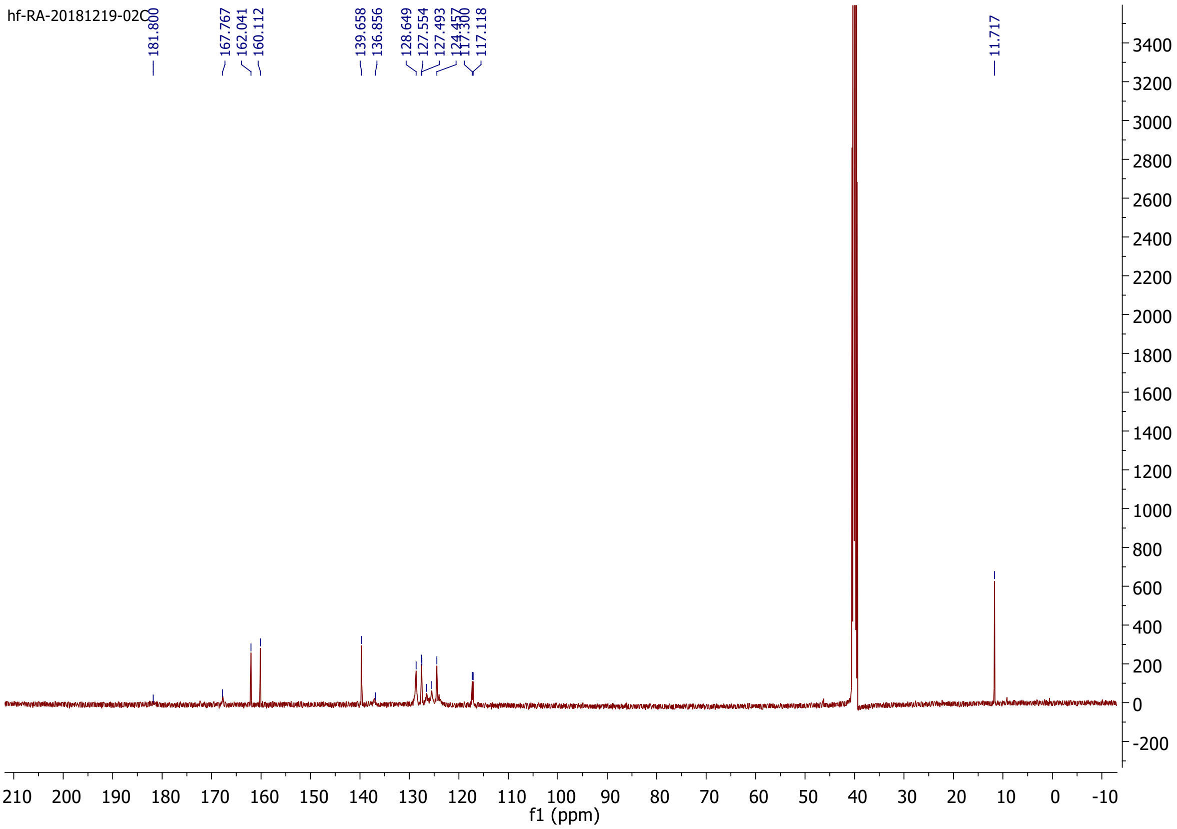

H[1] & ^{13}CNMR of Compound 4o

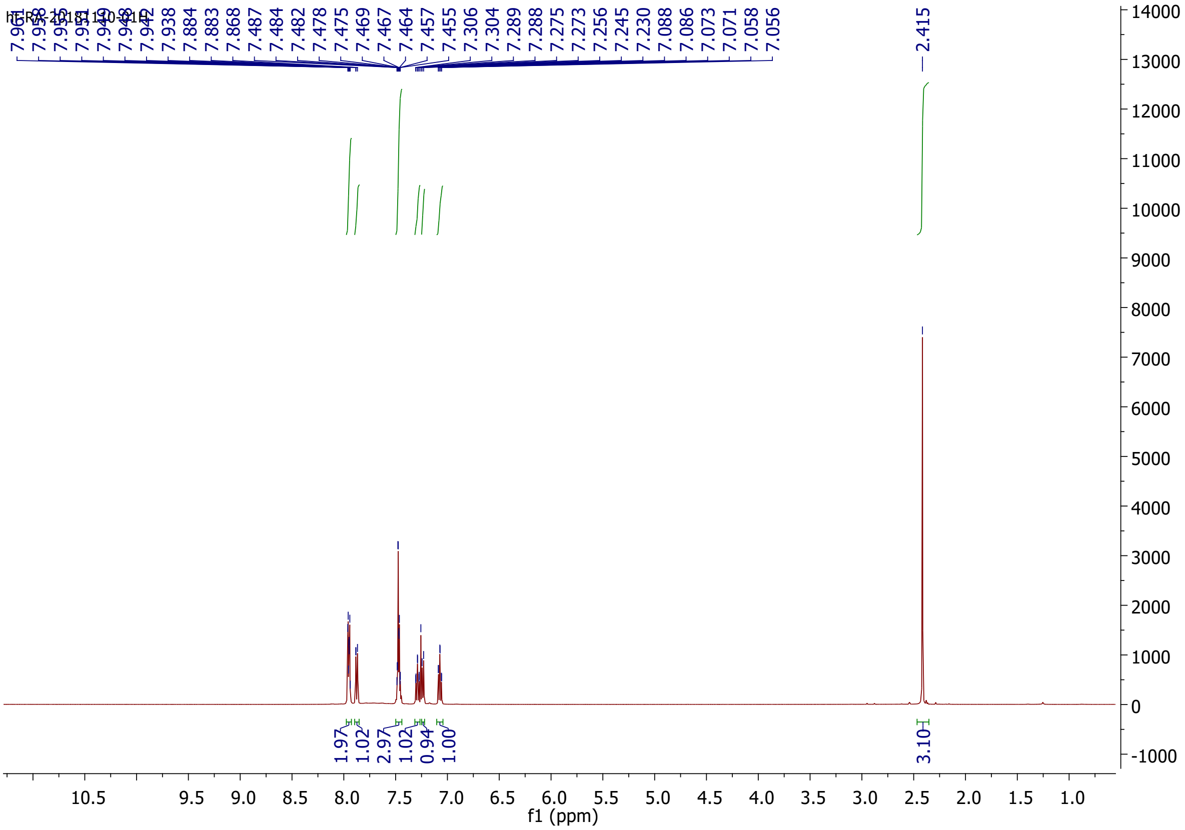

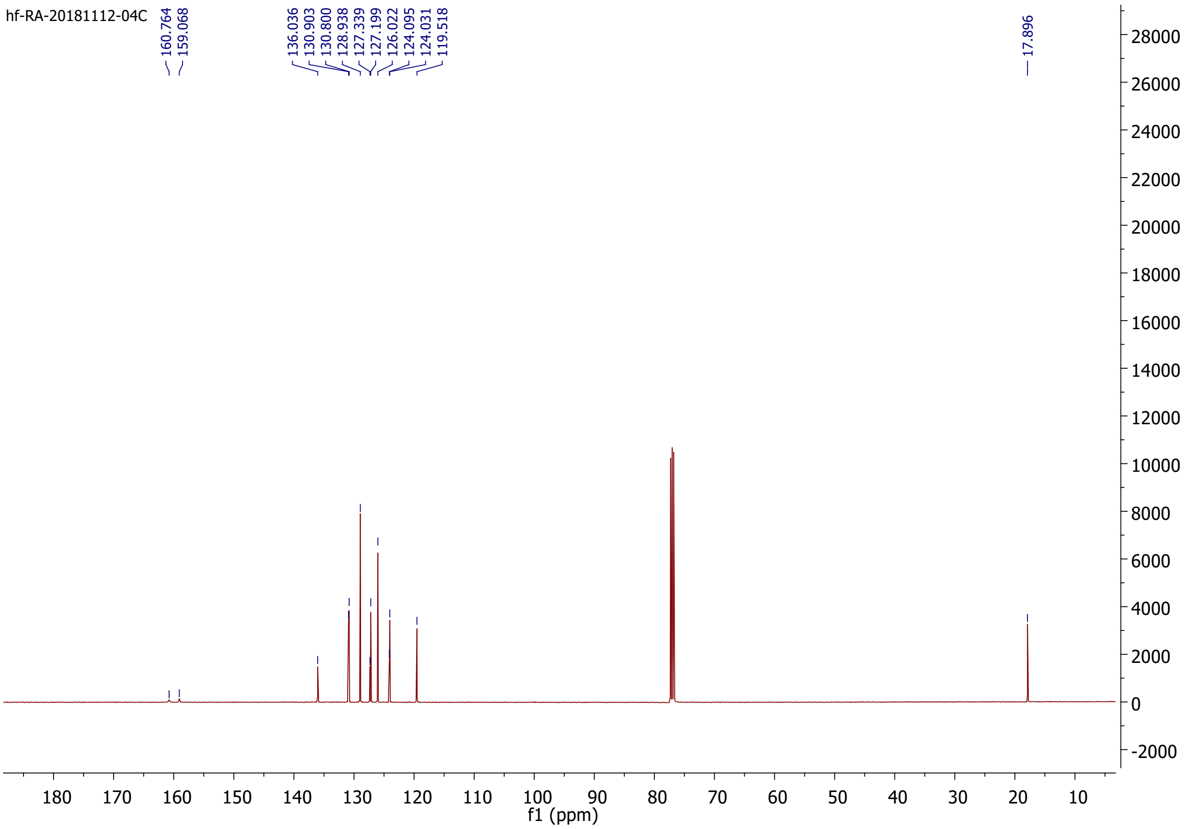

Supplement: RA-013-D3RA01953E-s001 [file RA-013-D3RA01953E-s001.pdf]
